# Six1 is essential for differentiation and patterning of the mammalian auditory sensory epithelium

**DOI:** 10.1371/journal.pgen.1006967

**Published:** 2017-09-11

**Authors:** Ting Zhang, Jinshu Xu, Pascal Maire, Pin-Xian Xu

**Affiliations:** 1 Department of Genetics and Genomic Sciences, Icahn School of Medicine at Mount Sinai, New York, New York, United States of America; 2 INSERM U1016, Institut Cochin, Paris, France; CNRS UMR 8104, Paris, France; Université Paris Descartes, Sorbonne Paris Cité, Paris, France; 3 Developmental and Regenerative Biology, Icahn School of Medicine at Mount Sinai, New York, New York, United States of America; Emory University School of Medicine, UNITED STATES

## Abstract

The organ of Corti in the cochlea is a two-cell layered epithelium: one cell layer of mechanosensory hair cells that align into one row of inner and three rows of outer hair cells interdigitated with one cell layer of underlying supporting cells along the entire length of the cochlear spiral. These two types of epithelial cells are derived from common precursors in the four- to five-cell layered primordium and acquire functionally important shapes during terminal differentiation through the thinning process and convergent extension. Here, we have examined the role of *Six1* in the establishment of the auditory sensory epithelium. Our data show that prior to terminal differentiation of the precursor cells, deletion of *Six1* leads to formation of only a few hair cells and defective patterning of the sensory epithelium. Previous studies have suggested that downregulation of Sox2 expression in differentiating hair cells must occur after *Atoh1* mRNA activation in order to allow Atoh1 protein accumulation due to antagonistic effects between Atoh1 and Sox2. Our analysis indicates that downregulation of Sox2 in the differentiating hair cells depends on Six1 activity. Furthermore, we found that Six1 is required for the maintenance of *Fgf8* expression and dynamic distribution of N-cadherin and E-cadherin in the organ of Corti during differentiation. Together, our analyses uncover essential roles of Six1 in hair cell differentiation and formation of the organ of Corti in the mammalian cochlea.

## Author summary

Auditory sensory hair cells and surrounding supporting cells are derived from common prosensory progenitors, which undergo rearrangements through intercalation to achieve extension and establish the mosaic structure between hair and supporting cells. Hair cells are susceptible to damage from a variety of insults and are unable to regenerate. Through temporal deletion of Six1 in the developing cochlea, we found that Six1 activity is crucial for proper hair cell fate specification and for the regulation and maintenance of the spatiotemporal pattern of Sox2, Fgf8 and E- and N-cadherins during differentiation. Our data uncover novel roles of Six1 in hair cell differentiation during the formation of the organ of Corti.

## Introduction

In response to a variety of signals, the prosensory progenitors in the floor of the mammalian cochlear duct enter terminal mitosis and then differentiate into a mosaic of mechanosensory hair cells (one row of inner and three rows of outer hair cells) interdigitated with several subtypes of nonsensory supporting cells, including inner border, inner phalangeal, inner and outer pillar and three rows of Deiters’ cells aligned in a medial-to-lateral direction. Failure to correctly produce or maintain these epithelial cells in the organ of Corti causes deafness. Understanding how hair cell morphogenesis is regulated has significant clinical implications, as hair cells are susceptible to damage from a variety of insults and are unable to regenerate.

The cochlea develops from the ventral portion of the otocyst, which elongates and begins to coil at E12 to reach a full 1.5 turns of the cochlear duct by E17.5 [1]. The prosensory progenitor cells proliferate to expand, and after reaching a defined number, exit the cell cycle from apex toward base between E12.5 to E14.5 to form a four- to five-cell layered non-proliferating precursor domain–the primordial organ of Corti, which is marked by expression of p27^Kip1^ [2, 3]. Soon after their cell cycle exit, the precursors initiate cell-type specific terminal differentiation near the base toward apex from E14.5 and undergo unidirectional cellular intercalation movement called convergent extension to form the two layers of epithelial cells, a lumenal layer of hair cells and a basal layer of supporting cells [3–5]. The Sox family transcription factor Sox2 is known to specify the precursor cells [6]. As distinct cell types undergo their specific differentiation in the precursor primordium, Sox2 shows a differential pattern of expression that is highly maintained in supporting cells through adulthood but downregulated in hair cells, which are induced by the basic helix-loop-helix (bHLH) transcription factor Atoh1 [7]. Current in vitro experimental evidence suggests that Atoh1 and Sox2 may have a mutually antagonistic relationship, in which Sox2 expression represses Atoh1-induced hair cell formation and expression of Atoh1 in hair cells leads to downregulation of Sox2 [8–11]. However, whether Atoh1 directly antagonizes Sox2 activity in vivo and how Sox2 is downregulated in the differentiating hair cells remain unclear. Moreover, despite extensive research on identifying factors that are important for hair cell morphogenesis, how these individual factors interact to generate different types of epithelial cells with distinct shapes and functions in the organ of Corti is still poorly understood. It is even more unclear how these interactions are precisely regulated to induce robust epithelial morphogenesis of the cochlea.

We have recently shown in the cochlear explant that Six1 of the homeodomain protein Six/So (Sine oculis) family interacts with Eya1 of the phosphatase-transcriptional coactivator Eya (Eyes absent) family to form a key transcriptional complex to activate *Atoh1* expression to induce a hair cell fate by interacting with Sox2 [11]. *Six1* is expressed in the otic placodal ectoderm as early as E8.75 and its expression becomes restricted to the ventral region of the otocyst where inner ear sensory organs form [12, 13]. The importance of *Six1* in inner ear development has been demonstrated by loss-of-function studies in mice and humans [12–15]. Mutations in the human *SIX1* gene cause sensorineural hearing loss [15], and the inner ear defects include either no or an undercoiled cochlea and absence or truncation of vestibular organs [16]. Loss of *Six1* in mice leads to an early arrest of inner ear development at the otocyst stage [12, 13]. More recently, a mouse model (*Catweasel*) carrying a novel point mutation (*Cwe*) within the Six1 homeodomain (p.E121G) has been identified through a large ENU mutagenesis screen and the *Cwe/Cwe* homozygous animals have severely truncated cochlea and semicircular canals [14]. Although the levels of Six1 expression have not been measured in *Cwe/Cwe* animals, the nature of the inner ear defects associated with this mutation indicates that it is a hypomorphic allele of *Six1*. During later stages of inner ear morphogenesis, strong *Six1* expression is maintained in the differentiating hair cells [12, 13]. However, despite the absolute necessity of *Six1* for inner ear development, it remains unknown how Six1 acts to drive sensory hair cell formation and the patterning of the organ of Corti.

In the present study, we used tamoxifen-inducible Cre mice to conditionally delete *Six1* after cochlea duct outgrowth to specifically investigate its potential role in auditory sensory epithelium development. Our data provides novel evidence to support a model in which Six1 serves as a critical factor for hair cell fate induction, differentiation and formation of the auditory sensory epithelium.

## Results

### *Six1* conditional mutant mice show defects in proliferation of the progenitor cells that give rise to the entire organ of Corti in the cochlea

To specifically investigate the role of *Six1* during the specification of sensory epithelial primordium in the developing cochlea, we used an inducible system to temporarily delete *Six1* after cochlear duct outgrowth by crossing the conditional *Six1*^*flox*^ mice [17] with *Eya1*^*CreER*^ [18] or *Sox2*^*CreER*^ [19] and administering tamoxifen from E11.5 to E12.5 before the sensory precursor cells exit cell cycle. Lineage tracing using *R26R*^*LacZ*^ reporter confirmed that one dose of tamoxifen administration at E11.5 induced *Eya1*^*CreER*^-lineage traced cells in the GER and all cells in the organ of Corti at P0 (S1A Fig) [20]. Similarly, previous studies have shown that tamoxifen treatment at E11.5 and E12.5 induced *Sox2*^*CreER*^-lineage traced cells in the GER and all cells in the organ of Corti as well as those in the vestibular organs [21]. Next, we confirmed whether tamoxifen administration at E11.5–12.5 specifically deletes Six1 function in only hair cell precursors or also supporting cell precursors using *Eya1*^*CreER*^ or *Sox2*^*CreER*^. Immunostaining with anti-Six1 revealed that Six1 is widely expressed in the cochlear epithelium at E12.5–13.5 (S1B Fig), but *Six1* CKO (*Eya1*^*CreER*^*;Six1*^*fl/fl*^ or *Sox2*^*CreER*^*;Six1*^*fl/fl*^) showed a significant reduction in the levels of Six1 within the sensory region 1–2 days after tamoxifen treatment (S1B Fig). At E17.5, Six1 antibody appeared to label not only hair cells but also supporting cells and the flanking GER/LER cells (S1C Fig), Six1 expression was lost in the hair cells in *Six1* CKO cochlea using either *Eya1*^*CreER*^ or *Sox2*^*CreER*^ as a deletor (S1D and S1E Fig). In addition, Six1 expression levels in some supporting cells adjacent to the GER also appeared to be reduced (S1D and S1E Fig). However, Six1 signal was strongly maintained in GER cells, some supporting cells and LER cells in the mutant, suggesting that the expression of Six1 in those precursor cells is activated before removal of Six1 and maintained even after tamoxifen induction. Based on these data, we conclude that both *Eya1*^*CreER*^ and *Sox2* ^*CreER*^ are able to specifically delete Six1 function in the hair cell precursors within the organ of Corti.

**Fig 1:**
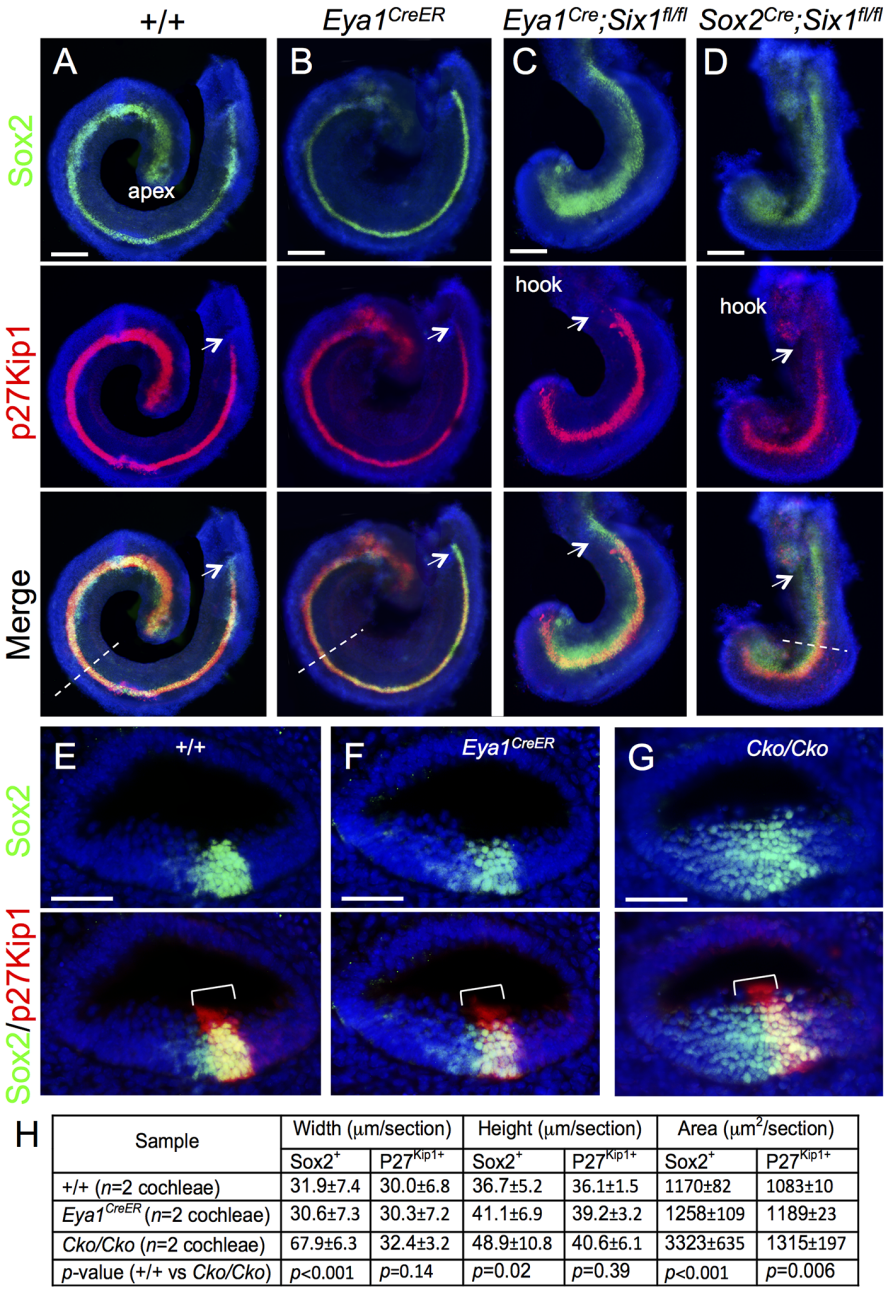
Deletion of *Six1* in the developing cochlea using *Eya1*^*CreER*^ or *Sox2*^*CreER*^ leads to shortened and thickened prosensory primordium. Cochleae were dissected from E14.5 embryos given tamoxifen at E11.5 (9 am) and E12.5 (9 am) and processed for whole-mount (A-D) or section (E-G) immunostaining with anti-Sox2 (green) and -p27^Kip1^ (red). Hoechst was used for nuclear-counter staining. (E-G) Section collected from mid-cochlear duct in wild-type, *Eya1*^*CreER*^ or *Sox2*^*CreER*^*;Six1*^*fl/fl*^ (*Cko/Cko*) littermates as indicated by dashed line in A, B, D respectively. Bracket indicates p27^Kip1^-positive prosensory domain within the cochlea epithelium and its width on mediolateral axis is comparable between control and mutant littermates. (H) Spatial calibration of Sox2^+^ and p27^Kip1+^ width, height and square area and value represents average number (±standard deviations) per section (6 μm) (see Methods for calibration). *P-*value was measured for +/+ and *Cko/Cko* using Two-tailed Student’s t-test. Scale bars: 200 μm in A-C and 40 μm in D,E.

To assess the effect of loss of *Six1* function between E11.5–12.5 on the establishment of the prosensory epithelial domain in the cochlea, we harvested inner ears from embryos at E14.5 and found that *Six1*^*Cko/Cko*^ (*Eya1*^*CreER*^*;Six1*^*fl/fl*^ or *Sox2*^*CreER*^*;Six1*^*fl/fl*^) inner ears were noticeably smaller in size compared to wild-type, *Eya1*^*CreER*^ or *Sox2*^*CreER*^ littermate controls (*n* = 6 embryos for each genotype; S2A Fig). We performed co-immunostaining of E14.5 cochleae with anti-Sox2 antibody to label all prosensory progenitors and p27^Kip1^ to mark postmitotic precursors in the nascent organ of Corti (Fig 1). In wild-type embryos, the cochlea had already reached more than one turn and most Sox2^+^ progenitors had exited the cell cycle to become p27^Kip1+^ along the entire cochlear duct (Fig 1A). In *Eya1*^*CreER*^ or *Sox2*^*CreER*^ littermates, the cochlea development appeared indistinguishable from that in wild-type controls at this stage (*n* = 6 embryos, Fig 1B). However, in *Six1* CKO littermates, shortening of the cochlear duct was evident at E14.5 (Fig 1C and 1D), and its length was comparable to that of the E12.5 control embryos [1]. While the Sox2^+^ domain expanded medially in *Six1* CKO samples along the length of the cochlear duct, most of Sox2^+^ cells still underwent cell-cycle exit to form the p27^Kip1+^ non-proliferating domain, which almost reached the base (Fig 1C). However, some *Six1* CKO embryos had fewer p27Kip1^+^ cells in the basal end (*n* = 5 out of 11 embryos, arrows in Fig 1D, compare to 1A-C). This suggests that not all prosensory progenitors in the base completed their cell-cycle exit to become non-proliferating precursors in the CKO. Immunostaining for Sox2/p27^Kip1^ on sections showed medially widened Sox2^+^ domain in the CKO (Fig 1E–1G). Statistical analysis of the width, height and area of Sox2^+^ and p27Kip1^+^ domain (*n* = 2 cochleae and 15 sections per cochleae) confirmed that the Sox2^+^ domain within the CKO cochlear epithelium is widened compared to that in littermate controls (Fig 1H).

**Fig 2:**
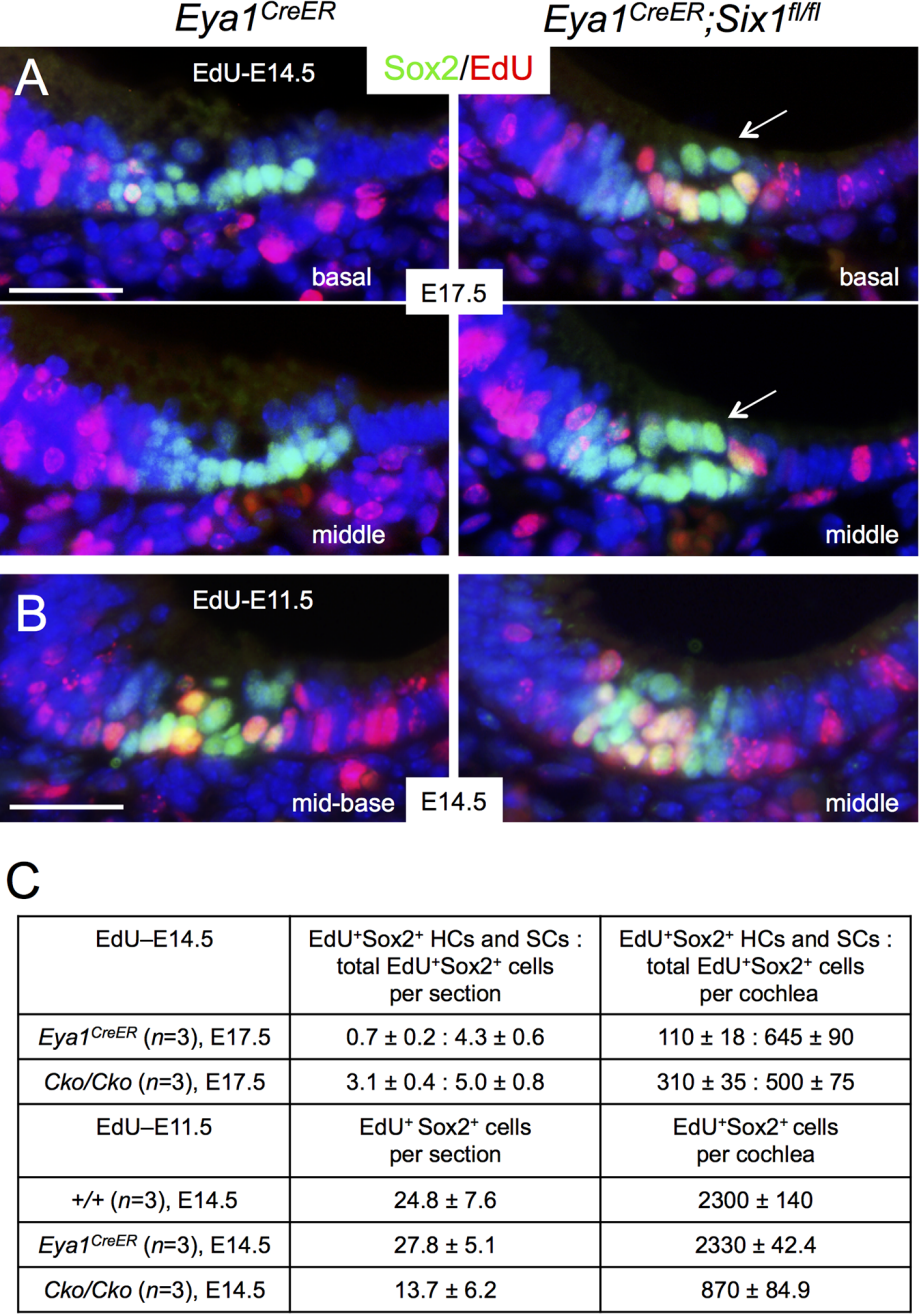
Altered cell proliferation in *Six1* CKO (*Eya1*^*CreER*^) cochlea epithelium. (A) Immunostaining for Sox2 (green) and EdU (red) on sections of basal and middle regions of cochleae from E17.5 embryos given EdU at E14.5 and tamoxifen at E11.5–12.5. Arrows point to high levels of Sox2 in cells within the lumenal layer. (B) Immunostaining for Sox2 (green) and EdU (red) on sections of middle region of cochleae from E14.5 embryos given tamoxifen at E11.5–12.5 and EdU at E11.5. (C) Number of EdU^+^Sox2^+^ cells per section (6 μm) or EdU^+^ cells per cochlea (see Methods for quantification). *P*-value was measured for +/+ and *Cko/Cko* using Two-tailed Student’s t-test: *p*<0.001 for E17.5 (EdU at E14.5) and *p* = 0.003 for E14.5 (EdU at E11.5). Scale bars: 30 μm in A,B.

To confirm that p27^Kip1^-negative Sox2^+^ progenitors in the base of the CKO cochlea are indeed proliferative progenitors, we injected the mitotic tracer 5-ethynyl-2’-deoxyurindine (EdU) at E14.5 and harvested inner ears at E17.5. Co-immunostaining for EdU and Sox2 and quantitative cell counting confirmed that there were more EdU-incorporated hair cells and supporting cells, majority of which was located in the base, in the CKO mutant than in control littermates (Fig 2A and 2C). More EdU-incorporated p27^Kip1+^ cells within the sensory epithelium were also observed in the base of *Six1* CKO cochlea (S2C and S2D Fig), while the *Six1* CKO inner ears were also smaller in size compared to littermate controls (S2B Fig). Together, these data suggest that there is a slight delay in the sensory epithelium development in the *Six1* CKO.

Next, we asked whether defective cochlear elongation between E12.5 and E14.5 prior to terminal differentiation of the precursor cells in *Six1* CKO mutant might be, at least in part, due to defective cell proliferation by co-injecting EdU together with tamoxifen at E11.5. Immunostaining and quantitative analysis indicated that EdU-incorporated Sox2^+^ cells were reduced to ~37% of those in the littermate controls at E14.5 (Fig 2B and 2C). TUNEL assay revealed that the number of apoptotic cells was only mildly increased in the CKO at E12.5–14.5 compared to the number in littermate controls (S3 Fig). Together, these data indicate that Six1 activity is necessary for normal cell proliferation of the epithelial progenitors and cochlear growth.

### Loss of *Six1* alters the spatiotemporal pattern of Sox2 expression during differentiation of the organ of Corti

**Fig 3:**
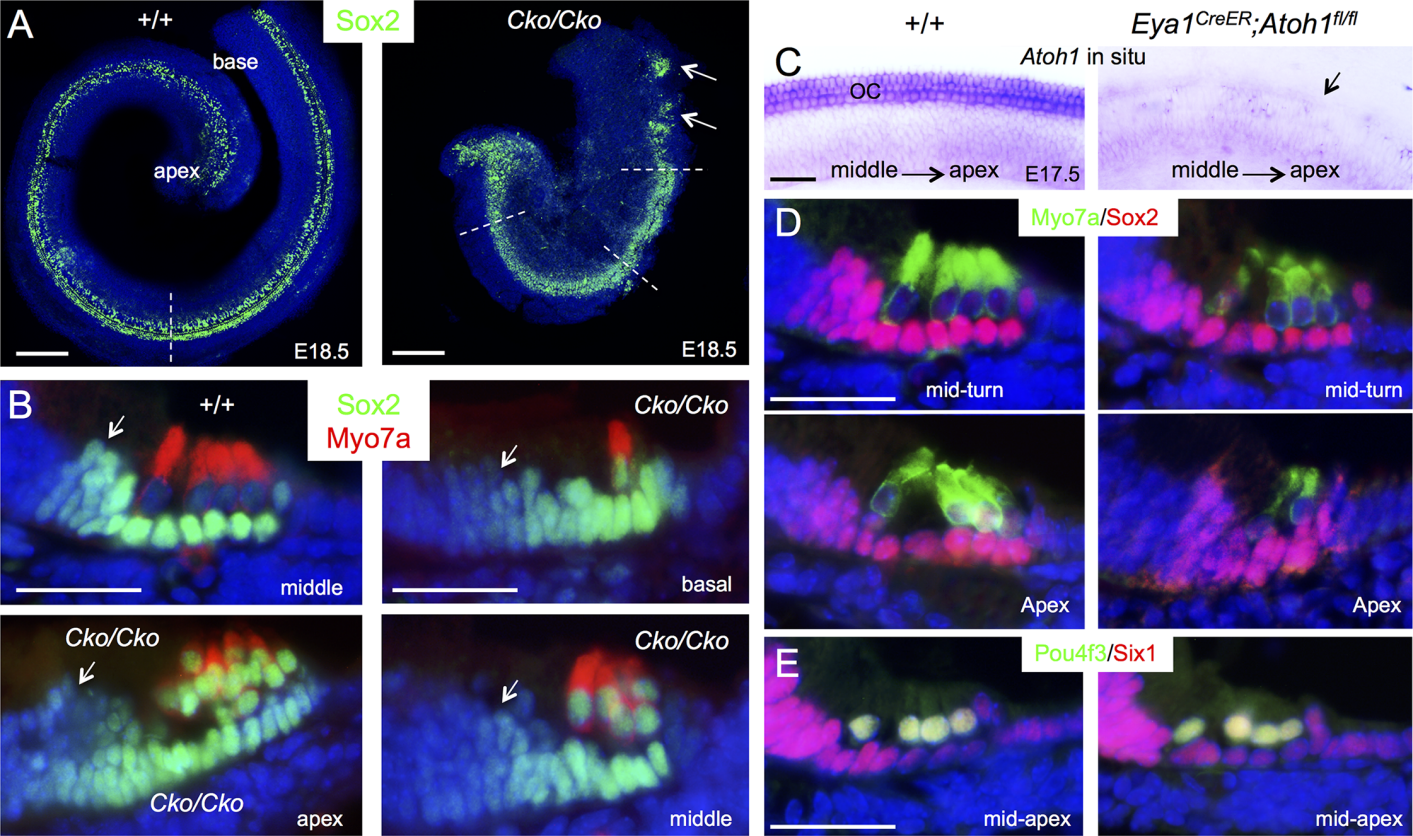
Downregulation of Sox2 in differentiating hair cells is disrupted in *Six1* CKO. (A) Immunostaining for Sox2 (green) on whole-mount and (B) Sox2 (green)/Myo7a (red) on sections of cochleae from wild-type or *Six1* CKO (*Eya1*^*CreER*^*;Six1*^*fl/fl*^) littermate embryos at E18.5 (given tamoxifen at E11.5–12.5). Cochlear section region was indicated by dashed line in A. Arrows point to Sox2 expression in the GER cells flanking the inner hair cells. (C) *Atoh1* in situ hybridization showing *Atoh1* expression in the organ of Corti in wild-type embryos at E17.5 and absence of *Atoh1* in *Atoh1*^*fl/fl*^*;Eya1*^*CreER*^ littermate embryos (arrow) given tamoxifen at E14.75–15.5. (D) Immunostaining for Myo7a (green) and Sox2 (red) on cochlear sections from E17.5 wild-type or *Eya1*^*CreER*^*;Atoh1*^*fl/fl*^ littermate embryos given tamoxifen at E14.75–15.5. (E) Immunostaining for Pou4f3 (green) and Six1 (red) on cochlear sections from E17.5 wild-type and *Eya1*^*CreER*^*;Atoh1*^*fl/fl*^ littermate embryos given tamoxifen at E14.75–15.5. Scale bars: 200 μm in A; 30 μm in B,D,E; 40 μm in C.

Sox2 specifies prosensory progenitors and is expressed in all progenitor cells at the early stages, but later during differentiation its expression is downregulated in hair cells and becomes restricted to the supporting cells in the organ of Corti [6, 8]. By E17.5, Sox2 levels in hair cells located in the basal and medial cochlear regions are normally downregulated in comparison to its high expression levels in the supporting cells (Fig 2A). However, we noticed that high levels of Sox2 are maintained in all cells within the *Six1* CKO organ of Corti (arrows, Fig 2A). This led us to speculate that Six1 may regulate the spatiotemporal pattern of Sox2 expression in the organ of Corti during differentiation. To rule out the possibility that the high levels of Sox2 in the cells within the lumenal layer in the CKO organ of Corti is due to developmental delay and confirm that those high Sox2^+^ cells are indeed hair cells, we harvested cochlea 1–2 days later at E18.5-P0 after tamoxifen administration at E11.5–12.5 and performed immunostaining for Sox2 and Myo7a, a marker specific for differentiating hair cells. While strong Sox2 expression is maintained in supporting cells through adulthood, relatively low Sox2 activity is detectable in GER (greater epithelial ridge) cells flanking the inner hair cells at E18.5 (Fig 3A and 3B). In *Six1* CKO, the cochlear duct not only appeared wider and thicker with discontinuation of Sox2^+^ domain in the base compared to that in wild-type controls (arrows, Fig 3A; *n* = 6), but also was shortened to 0.75- to 1-turn. Along with cochlear elongation between E14.5 to E18.5, hair cell differentiation occurs near the mid-base and reaches the basal end and apex in a medial-to-lateral gradient to form one row of inner and three rows of outer hair cells along the entire length of the cochlea by E18.5 as marked by Myo7a (Fig 3B). The hair cells are interdigitated by distinct subtypes of specialized supporting cells: one row of inner border cells and one row of inner phalangeal cells surrounding the inner hair cells, two rows of pillar cells (one row of inner and one row of outer pillar cells) lining the space between the inner and outer hair cells–the tunnel of Corti–and three rows of Deiters’ cells associated with the outer hair cells. In *Six1* CKO cochlea, Myo7a^+^ hair cells were indeed present but they appeared irregularly with only one cell toward the base and more than four cells toward the apex. However, high levels of Sox2 expression were still maintained in Myo7a^+^ hair cells, as in the supporting cells that also appeared to be aligned irregularly. Furthermore, lower Sox2 activity appeared to expand medially into GER cells in the mutant (arrows, Fig 3B).

The observation of high levels of Sox2 expression in *Six1* CKO hair cells was a surprising finding because it has been argued that Atoh1 is involved in the downregulation of Sox2. To test this further, we examined the dependence of Sox2 levels on Atoh1 in hair cells by deleting *Atoh1* from E14.5–15.5 using *Eya1*^*CreER*^. In situ hybridization of cochlea at E17.5 confirmed deletion of *Atoh1* in the differentiating hair cells (Fig 3C). However, immunostaining for Sox2, Six1 and Pou4f3 revealed no detectable changes in *Atoh1* CKO (Fig 3D and 3E), which was consistent with previous observations detected by western blot and in situ hybridization for these genes [22]. Thus, deletion of *Atoh1* in differentiating hair cells does not lead to upregulation of Sox2. Based on these data, we conclude that Six1 activity is crucial for downregulation of Sox2 in the differentiating hair cells during cochlear development.

### *Six1* is required for hair cell fate specification in the cochlea

**Fig 4:**
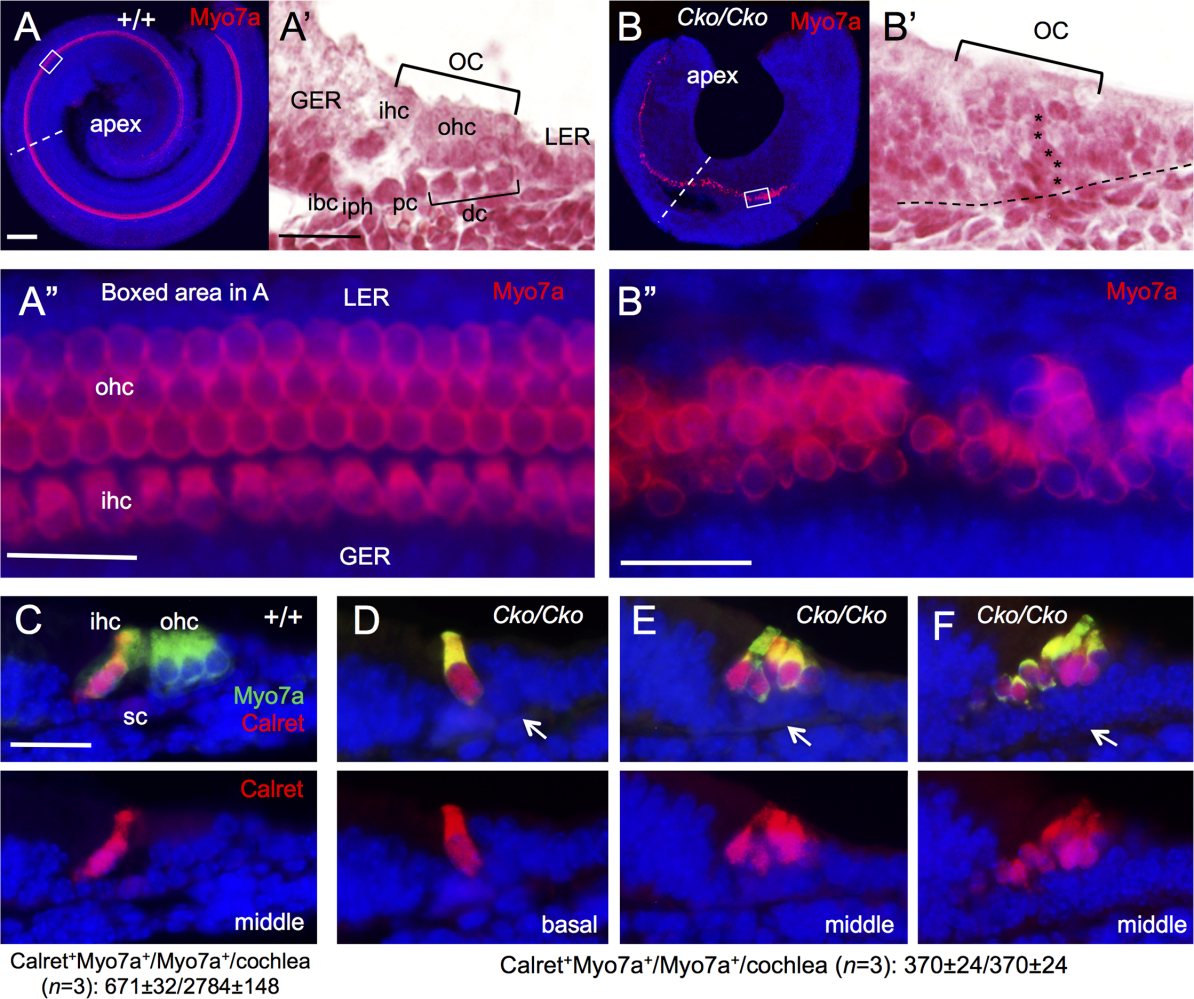
Lack of outer hair cell formation in *Six1* CKO (*Eya1*^*CreER*^) cochlea. (A,B) Immunostaining for Myo7a of cochlea from E18.5 embryos given tamoxifen at E11.5–12.5. (A’,B’) H&E staining of section from middle region of cochlea indicated by dashed line in A or B respectively. (A”,B”) Higher magnification of boxed region in A or B respectively. (C-F) Co-immunostaining on sections for Myo7a (green) and Calretinin (red). Abb.: dc, Deiters’ cells; GER, greater epithelial ridge; ibc, inner border cell; ihc, inner hair cells; iph, inner phalangeal cell; oc, organ of Corti; ohc, outer hair cells; pc, pillar cells; sc, supporting cells. Scale bars: 200 μm in A and B; 50 μm in A’,B’ and C-F; 30 μm in A” and B”.

As inner hair cells differentiate prior to outer hair cells, we next sought to characterize whether the Myo7a^+^ hair cells observed in *Six1* CKO cochlea are inner hair cells, outer hair cells or both. Whole-mount immunostaining of cochlea at E18.5 revealed one row of inner and three rows of outer hair cells along the entire length of the cochlea in wild-type control (Fig 4A and 4A”). In the *Six1* CKO, while the length of cochlea was shortened to ~0.75- to 1-turn, Myo7a^+^ cells extended to the apical end but were missing in the base (*n* = 6; Fig 4B). This suggests that hair cell differentiation toward the basal end fails to occur.

Higher magnification analysis showed that hair cells that had formed in *Six1* CKO had abnormal morphology and irregular alignment with an uneven numbers of hair cells on the mediolateral axis, ranging from one to multiple cells (Fig 4B”). As seen on sections, the organ of Corti consists of two layers of epithelial cells, a lumenal layer of hair cells and a basal layer of supporting cells (Fig 4A’) flanked by nonsensory epithelial cells in the GER/LER (greater/lesser epithelial ridge). In contrast, *Six1* CKO organ of Corti is retained as a four- to five-cell layered epithelium (Fig 4B’), which is almost comparable to the non-proliferating precursor domain in E14.5 control embryos. This clearly indicates a defect during terminal differentiation of the p27^Kip1+^ precursor cells in the mutant. Analysis of vestibular sensory organs showed largely reduced utricular and saccular macula with fewer hair cells and no hair cells in crista ampullaris in all three semicircular canals (S4 Fig).

Interestingly, all Myo7a^+^ cells in *Six1* CKO cochlea are positive for Calretinin (Fig 4D–4F), a marker specific for inner hair cells (Fig 4C), suggesting that the hair cells developed in the CKO cochlea treated with tamoxifen between E11.5–12.5 might be inner hair cells. Quantitative counting revealed that the total number of hair cells in *Six1* CKO cochlea (Myo7a^+^Calretinin^+^ cells/total Myo7a^+^ cells = 370±24/370±24 per cochlea; *n* = 3) decreased to ~53% of the total number of inner hair cells in wild-type cochlea (Calretinin^+^Myo7a^+^ cells/total Myo7a^+^ cells = 671±32/2784±148 per cochlea; *n* = 3). Thus, the absence of outer hair cell formation in *Six1* CKO is not likely due to a conversion of outer hair cells to inner hair cells, but rather caused by a failure of activation of the outer hair cell differentiation program.

**Fig 5:**
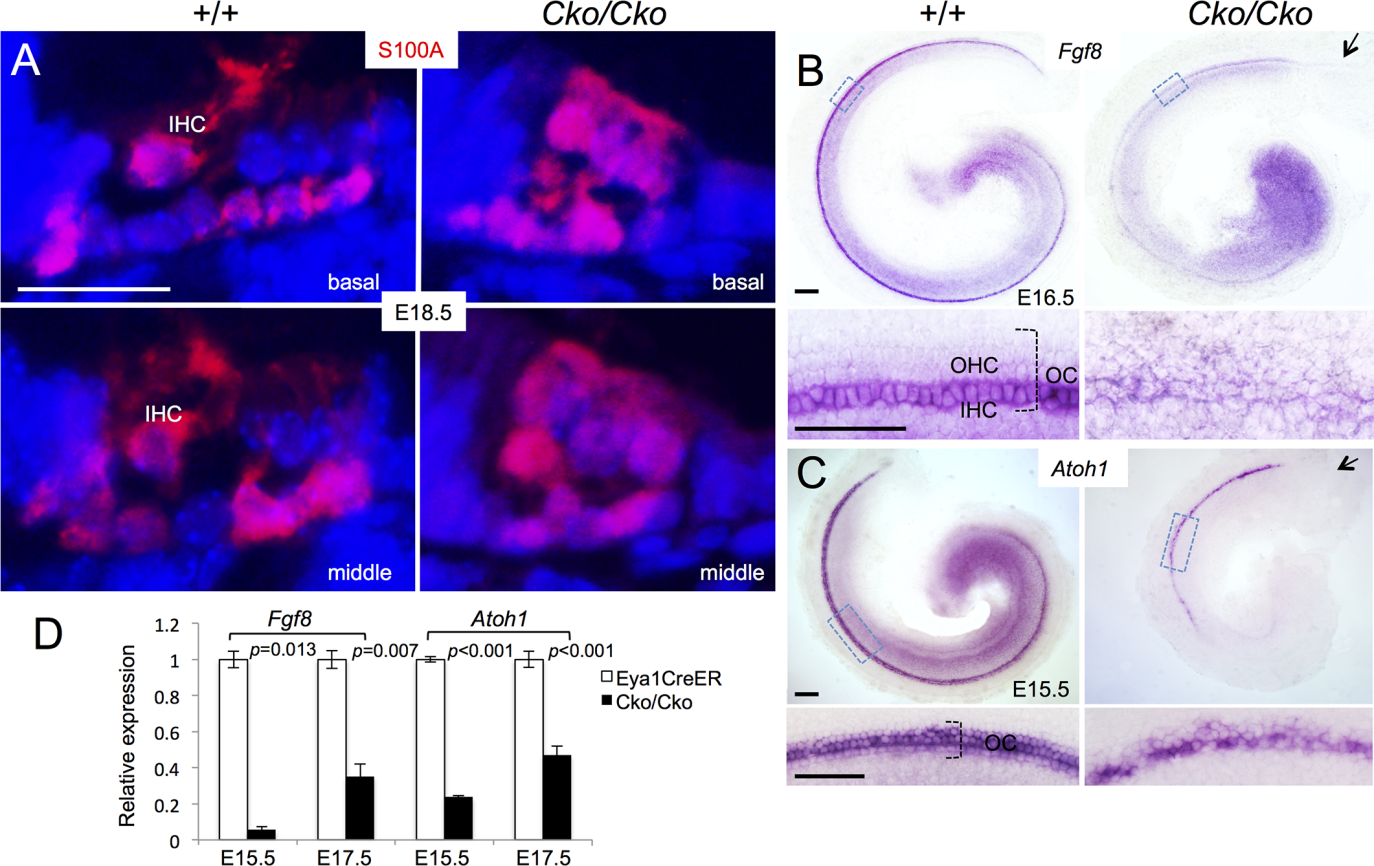
Reduced *Fgf8* expression in *Six1*^*Cko/Cko*^ mutant (*Eya1*^*CreER*^*;Six1*^*fl/fl*^ given tamoxifen from E11.5–12.5). (A) Immunostaining for S100A (red) on sections of cochleae from E18.5 wild-type or *Six1*^*Cko/Cko*^ littermate embryos. (B) Whole-mount in situ hybridization with *Fgf8* ribo-probe showing *Fgf8* expression in inner hair cells (IHC) in wild-type control littermates and decreased expression in remaining hair cells in *Six1*^*Cko/Cko*^ littermates at E16.5. Lower panels are higher magnification of boxed areas. (C) Whole-mount in situ hybridization of E15.5 cochleae showing *Atoh1* mRNA expression in the hair cells in *Six1*^*Cko/Cko*^ cochlea. Arrow in B,C points to lack of *Fgf8* or *Atoh1* expression in the basal end of cochlea in *Six1*^*Cko/Cko*^. Lower panels are higher magnification of boxed areas. (D) *Fgf8* and *Atoh1* mRNA expression were examined by qRT-PCR in *Eya1*^*CreER*^ and *Six1* CKO littermates at E15.5 and E17.5 (given tamoxifen at E11.5–12.5). Two-tailed Student’s t-test was used for statistical analysis. Scale bars: 20 μm in A,B, 100 μm in upper panels of C,D and 50 μm in lower panels of C,D.

To further confirm our observation, we immunostained for the calcium-binding protein S100A, which labels inner hair cells, inner phalangeal cells and Deiters’ cells [23, 24] (Fig 5A). In *Six1* CKO cochlea, S100A labeled not only all hair cells but also all supporting cells. This further suggests that the remaining hair cells in the CKO might be inner hair cells.

In situ hybridization with *Fgf8* riboprobe, a marker specific for inner hair cells [25], revealed strong *Fgf8* expression in the inner hair cells in E16.5 cochlea (Fig 5B). However, *Fgf8* expression was decreased in *Six1* CKO littermates and appeared in the remaining hair cells at E16.5 (Fig 5B). Quantitative real-time RT-PCR (qRT-PCR) confirmed a large reduction of *Fgf8* expression in the mutant at E15.5 and E17.5 (Fig 5D). Together, these results suggest that Six1 may regulate the maintenance of *Fgf8* expression in the inner hair cells.

In situ hybridization confirmed that *Atoh1* mRNA is expressed in the hair cells of *Six1* CKO cochlea at E15.5, at which *Atoh1* expression has not yet reached its apex in both controls and *Six1* CKO littermates (Fig 5C). However, its expression levels appeared to be lower in the CKO than in the control littermates (Fig 5C), and this reduction was confirmed by qRT-PCR (Fig 5D). Thus, while *Atoh1* expression is induced in the postmitotic precursors, which only differentiate into inner hair cells in *Six1* CKO treated with tamoxifen between E11.5–12.5, Six1 may play a role in controlling the maintenance or upregulation of *Atoh1* in hair cells during differentiation.

**Fig 6:**
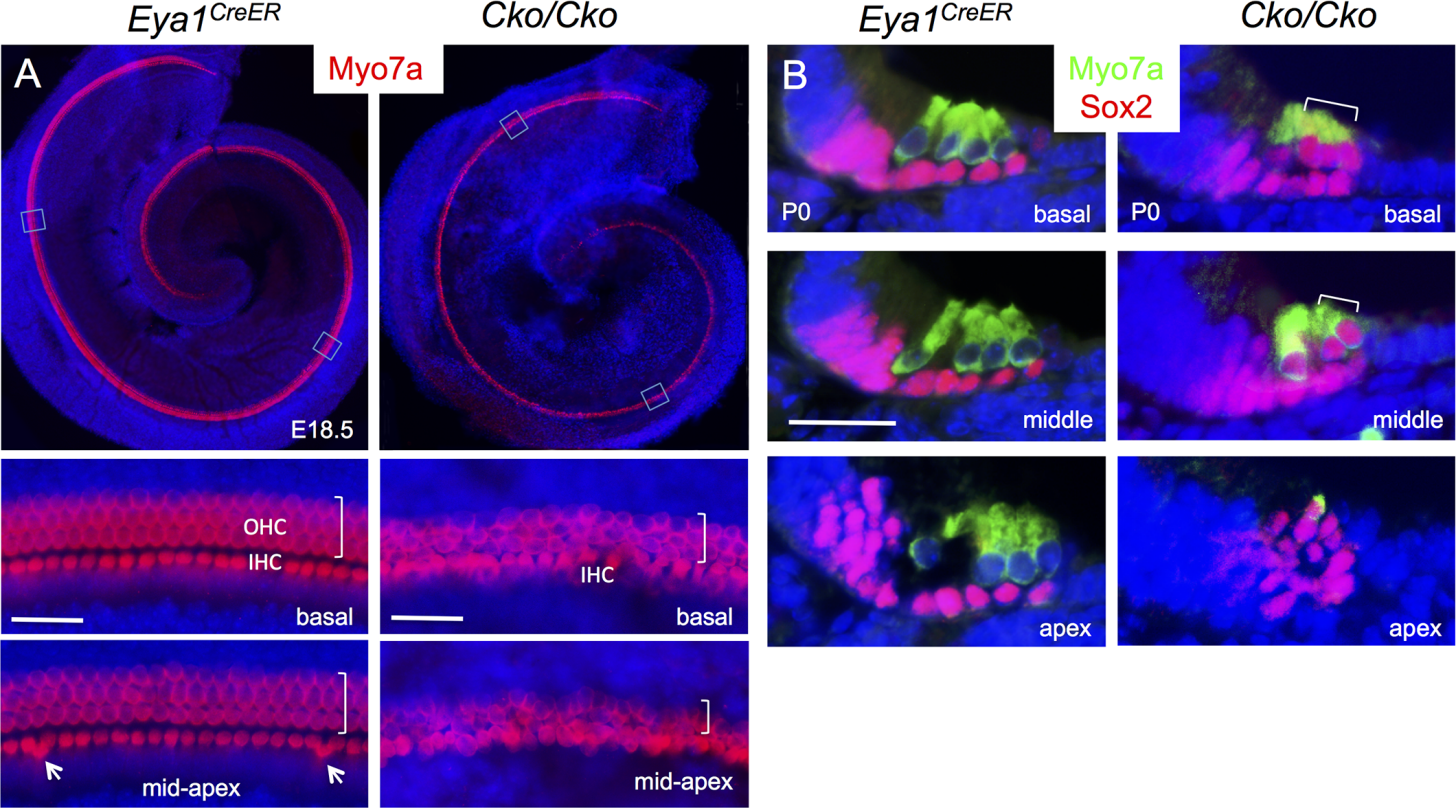
Temporal deletion of *Six1* between E12.75–13.5 blocks hair cell induction. (A) Whole-mount Myo7a staining of E18.5 cochleae showing four rows of hair cells along the entire cochlea in *Eya1*^*CreER*^ mice but extra inner hair cells present from middle toward apex (arrows). In *Six1* CKO cochlea, the organ of Corti appears narrower and hair cells show abnormal morphology with gradually decreased outer rows of hair cells from four in the base and one row in the apex. (B) Co-immunostaining for Myo7a and Sox2 on sections from P0 cochleae treated with tamoxifen between E13.5–14.5 showing one inner and three outer hair cells in the base, one inner and two outer hair cells in the middle and only one hair cells in the apex in *Six1* CKO. All Myo7a^+^ hair cells are also Sox2^+^ in the CKO mutant. Scale bars: 30 μm.

As outer hair cells differentiate more than one day after the onset of inner hair cell differentiation, we next sought to further clarify our observation by administering tamoxifen more than one day later between E12.75-E13.5 or E13.5–14.5 to examine whether outer hair cells also form in the mutant. Analysis of E18.5 cochleae treated with tamoxifen between E12.75–13.5 showed that the *Six1* CKO cochlea reached a full 1.5 turns, and that four rows of hair cells formed in the basal turn, but revealed a pattern of severity that parallels the normal process of hair cell differentiation, with outer rows more affected than inner rows in the medial turn, and no outer hair cells in the apex (*n* = 6, Fig 6A). A similar observation was obtained in P0 cochleae treated with tamoxifen between E13.5–14.5 (*n* = 6, Fig 6B). All hair cells formed in *Six1* CKO cochlea also displayed abnormal morphology and maintained high levels of Sox2 (Fig 6B). These data provide additional evidence that Six1 activity is necessary for hair cell fate specification and downregulation of Sox2 in the differentiating hair cells.

### Alteration in cell morphology and distribution of cadherins in *Six1* CKO organ of Corti

**Fig 7:**
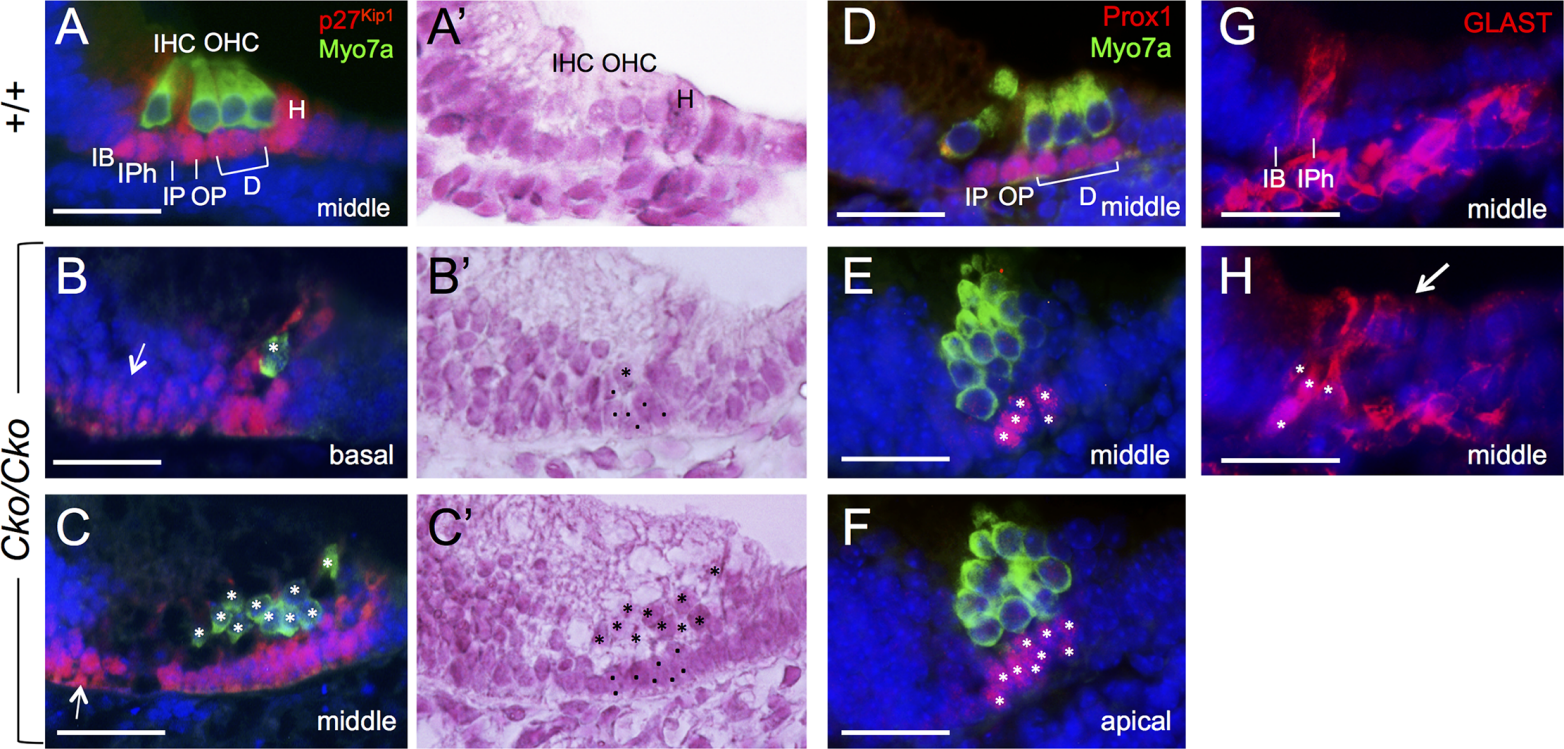
Differentiation and misalignment of supporting cell subtypes in the absence of *Six1*. Antibody labeling for Myo7a for hair cells and p27^Kip1^ (A-C), Prox1 (D-F) and GLAST (G,H) for supporting cells in the organ of Corti in wild-type and *Six1* CKO (*Eya1*^*CreER*^*;Six1*^*fl/fl*^) littermates given tamoxifen from E11.5–12.5. A’,B’,C’ are A.B,C counter-stained with hematoxylin respectively. Arrow in B,C points to expansion of p27^Kip1^ expression in the GER cells. Asterisks in B,B’,C,C’ point to remaining hair cells and dots point to supporting cells. Asterisks in E,F,H point to supporting cells. Abb.: D, Deiters’ cells; H, Hensen’s cells; IB, inner border cell; IHC and OHC, inner and outer hair cells; IPh, inner phalangeal cells; IP and OP, inner and outer pillar cells. Scale bars: 30 μm.

We failed to observe S100A-negative pillar cells on all sections from the *Six1* CKO cochleae (*n* = 3, Fig 5A), suggesting that the pillar cells are not formed in the mutant. We therefore further investigated whether loss of *Six1* also results in loss of supporting cell subtypes, using specific marker gene analysis. Despite irregular shape, all Sox2^+^ supporting cells underlying the hair cells in *Six1* CKO cochlea were positive for p27^Kip1^ at E18.5 (*n* = 5, Fig 7B and 7C), whose expression is normally restricted to all supporting cells, including Hensen’s cells flanking the outermost outer hair cells (Fig 7A). Similar to Sox2, p27^Kip1^ expression also showed medial expansion to the flanking GER cells (arrows, Fig 7B and 7C, *n* = 3). However, those cells expressed neither hair cell markers nor other supporting cell markers. For example, Prox1, which is expressed in pillar cells and Deiters’ cells (Fig 7D) and thought to act downstream of Sox2 [8], was found to be expressed in supporting cells underlying the hair cells but not in adjacent GER cells in *Six1* CKO cochlea at E18.5 (*n* = 4, Fig 7E and 7F). However, the Prox1^+^ cells in the CKO failed to align into a characteristic one-cell layer, and there were five or more in the basal to middle region (Fig 7E) and up to ten Prox1^+^ cells underlying the two- to three-cell layered hair cells toward the apex (Fig 7F). Inner border and inner phalangeal cells labeled by Glutamate-aspartate transporter (GLAST) (Fig 7G) were detectable in the medial region of the organ of Corti in *Six1* CKO (*n* = 4). However, in contrast to their apical process that only surrounds the inner hair cells in the wild-type littermates (Fig 7G), these GLAST^+^ cells in the mutant appeared to make apical process that surrounded all hair cells (arrow, Fig 7H).

**Fig 8:**
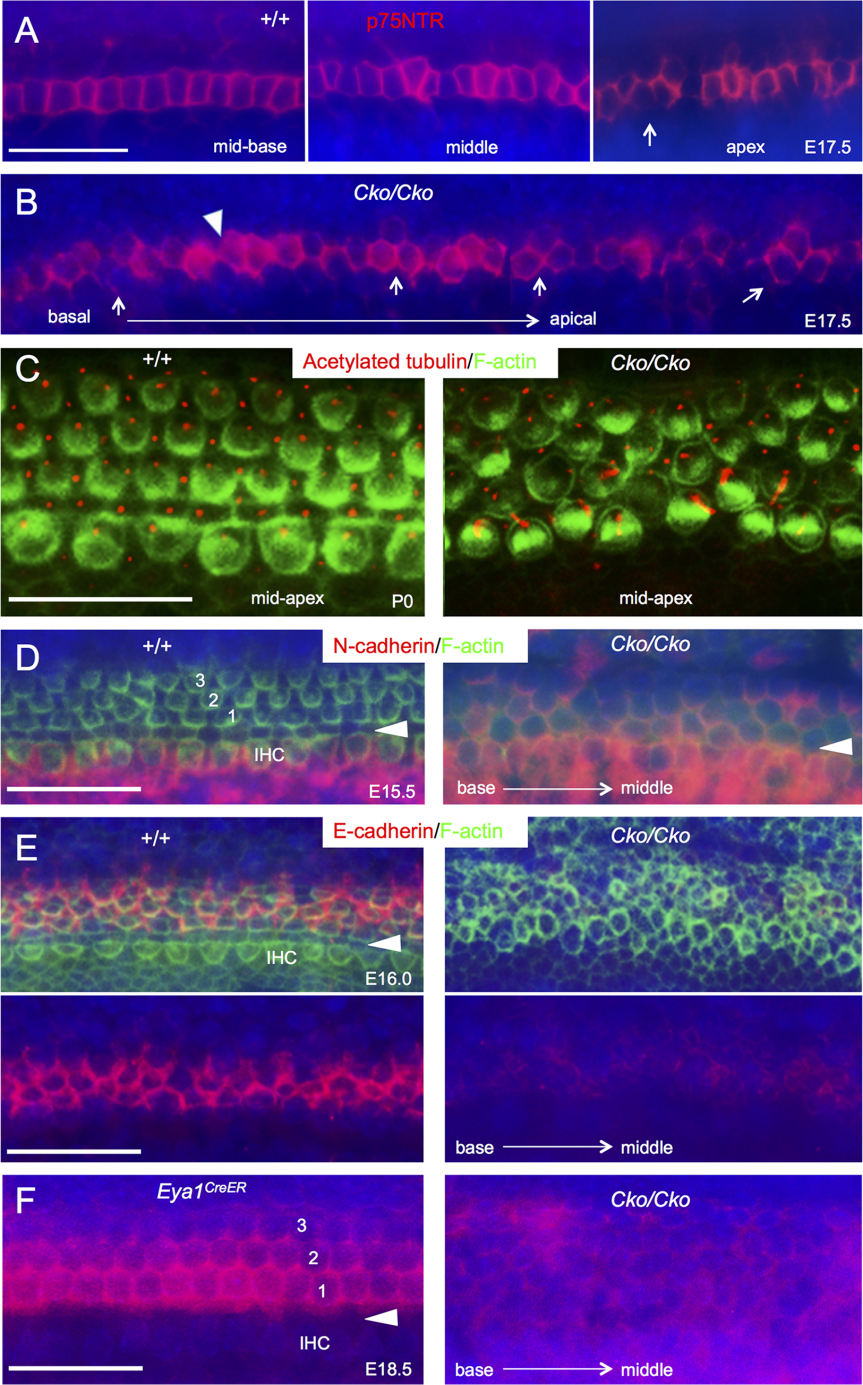
Alteration in cellular morphology and distribution of N- and E-cadherin in *Six1* CKO organ of Corti (tamoxifen at E11.5–12.5, *Eya1*^*CreER*^). (A,B) Inner pillar cells labeled by p75^NTR^ in wild-type cochlea and *Six1* CKO littermate cochleae at E17.5. Arrow in A points to dynamic remodeling of cellular contacts toward the less differentiated apical region. Arrows and arrowhead in B point to three or more inner pillar cells in contact and cellular contact shrinkage respectively in *Six1* CKO organ of Corti. (C) F-actin (phalloidin) and acetylated tubulin staining showing alteration in cell orientation and alignment in *Six1* CKO at P0. (D-F) Lumenal surface views of the cochleae from wild-type, *Eya1*^*CreER*^, and *Six1* CKO (tamoxifen at E11.5–12.5, *Eya1*^*CreER*^) (D,E) embryos at E15.5 (D), E16.0 (E) or E18.5 (F). Samples were stained for phalloidin and N-cadherin (D) or E-cadherin (E,F). Arrowheads mark the separation between inner hair cells and outer hair cells. The developing organ of Corti is identified by cellular morphology and cortical actin enrichment in the nascent hair cells. Scale bars: 30 μm A,B, 20 μm C, and 30 μm E-F.

As Prox1 is expressed in both inner and outer pillar cells, we next used inner pillar cell specific marker p75^NTR^ to further clarify the presence of inner pillar cells in the CKO. Indeed, inner pillar cells labeled by p75^NTR^ were present but showed changes in cell shape and cell contacts on the lumenal surface. During cochlear elongation, rearranging epithelial cells shrink junctions that are oriented perpendicular to the axis of extension and subsequently resolve such shrinkage to restore more isodiametric shapes. In control animals at E17.5, inner pillar cells in the basal region are aligned in a single row with stable cellular contacts (long junction) (Fig 8A). Dynamic remodeling of cellular contacts was seen toward the less differentiated apical region (arrow in Fig 8A). In *Six1* CKO, the inner pillar cells showed changes in morphology and there were three or more inner pillar cells in contact (arrows, Fig 8B, *n* = 5) and cellular contact shrinkage (arrowhead, Fig 8B) throughout the entire cochlea, indicating a clear defect in cellular rearrangement in the absence of *Six1*.

Further examination also uncovered morphological alteration in hair cells in *Six1* CKO. During differentiation, hair cells form actin-rich V-shaped stereociliary bundle with graded heights that are all individually aligned and point in the same direction toward the lateral side of the organ of Corti. This polarization process is initiated by the migration of the centrally positioned kinocilium to the periphery from ~E16.5 [26]. At P0, the uniform orientation of hair cells and their interdigitation with nonsensory supporting cells on the lumenal surface with asymmetric and lateral distribution of kinocilium as outlined by F-actin and acetylated tubulin staining respectively were evident throughout the entire organ of Corti in wild-type controls (*n* = 6, Fig 8C). However, in absence of *Six1* function, we found that individual hair cell orientation was severely affected (*n* = 6, Fig 8C). Collectively, our results demonstrate that absence of *Six1* not only limits the normal extension of the cochlear duct, but also results in significant defects in cell shape within the plane of cochlear sensory epithelial sheet during terminal differentiation.

As selective cell adhesion, mediated by cadherins, plays a pivotal role in regulating the shape and topology of the cells in tissue morphogenesis [27], we therefore tested whether loss of *Six1* leads to changes in adhesion by comparing the distributions of cadherins in controls and *Six1* CKO littermates. At E15.5, the organ of Corti has differentiated in the base and middle, and consists of one row of inner and three rows of outer hair cells interdigitated with nonsensory supporting cells as outlined by phalloidin staining (Fig 8D). N-cadherin is expressed in the cochlea from E14.0 [28] and its distribution is restricted to cells medial to the outer hair cell from E15.5 (Fig 8D). In *Six1* CKO, the general integrity of the organ of Corti is not maintained and as expected, due to the expanded (more than one-cell) layer of hair cells (Fig 4B’ and 4B”), the organization between hair cells and their interdigitated nonsensory supporting cells is apparently disrupted, similar to that observed at P0 (Fig 8C). In *Six1* CKO, N-cadherin expression was observed in the medial region, but its expression expanded to the lateral region of the organ of Corti (*n* = 5, Fig 8D). In contrast, E-cadherin is normally restricted to the region of outer hair cells and the region lateral to it from E16.0 (Fig 8E), and its onset of membrane localization coincides with the stabilization of cell junctions in the region lateral to the inner pillar cells. However, the levels of E-cadherin at the cell membrane were reduced in *Six1* CKO cochlea at E16.0 (*n* = 4, Fig 8E). By E18.5, relatively lower levels of E-cadherin at the cell membrane were widely detectable in all cells within the sensory epithelium as well as in the GER cells in the CKO (*n* = 4, Fig 8F). These results indicate that loss of *Six1* function alters the patterns of N- and E-cadherin distribution in the cochlea and the structure of the organ of Corti.

## Discussion

The role of *Six1* in inner ear development has been previously investigated [12–14]. However, due to lack of inner ear formation beyond the otocyst stage in *Six1*-null mice, the molecular modules carried out by *Six1* in the auditory sensory epithelium remain unknown. In cochlear explant, we have shown that Six1 forms a complex with Eya1 and Sox2 to activate *Atoh1* expression to induce hair cell fate in the GER cells [11]. Here, we for the first time investigated the in vivo requirement of *Six1* in auditory sensory epithelium specification and differentiation. Our analyses indicate that Six1 is crucial not only for proper fate specification but also for proper patterning of the precursor cells in the auditory sensory epithelium, which are necessary steps for the formation of the organ of Corti in the cochlea.

Previous studies have shown that, in the otocyst, Six1 promotes both proliferation and survival of the otic epithelial cells [12, 13]. However, Six1 activity does not appear to be crucial to cell survival in the developing cochlea as no significant difference in apoptosis was observed in *Six1* CKO. Reduced proliferation in *Six1* CKO cochlear epithelium suggests that Six1 plays a critical role in maintaining the prosensory progenitor cells at proliferative state in order to expand to a certain number, which explains shortened cochlea and a reduced number of hair cells observed in *Six1* CKO. How does Six1 act to regulate cell proliferation? We found that Six1 forms a complex with Eya1, N-Myc and c-Myc proteins in E12.5–13.5 cochlea [11, 29]. Myc proteins are known to be important for cell proliferation and growth, making it plausible to speculate that Six1 works together with its cofactors such as Eya1 and Myc proteins to regulate cell proliferation and growth. Defects in cell division and growth before terminal mitosis are likely to lead to shortened cochlea occurring in the *Six1* CKO mutant.

**Fig 9:**
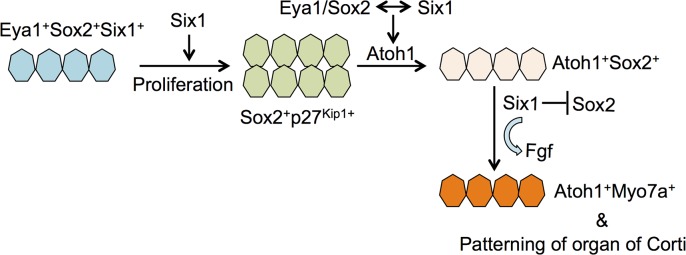
Model illustrating the role of Six1 in regulating auditory sensory cell development. This study demonstrates that Six1 regulates proliferation of sensory progenitors in the cochlea epithelium. While a direct interaction between Eya1/Six1/Sox2 proteins coordinately regulates Atoh1 expression in cochlear explant, this study provides in vivo evidence supporting a role for Six1 in hair cell fate specification and *Atoh1* expression. In differentiating hair cells, our data show that Six1 activity is necessary for downregulation of Sox2 and maintenance of *Fgf8* expression.

Our observation of the absence of outer hair cell formation in the *Six1* CKO mutant provides the first in vivo evidence supporting a model in which Six1 serves as a key factor for hair cell fate specification (Fig 9). In cochlear explants, we found that Six1 forms a complex with Eya1 and Sox2 to synergistically activate *Atoh1* to induce hair cell fate [11]. Given that Eya1 is a transcriptional coactivator interacting with DNA-binding proteins Six1 and Sox2 to transactivate *Atoh1* expression, and the latter (Sox2) is necessary for *Atoh1* activation in vivo [6, 11, 30], we have previously proposed a model in which Eya1 bridges Sox2 and Six1 to undergo protein-interaction-dependent and binding sequence-dependent conformational changes to form a compact and active complex capable of transcriptional activation of *Atoh1* [11]. Based on this model, if all three genes are necessary for *Atoh1* activation in vivo, deletion of any one of the three genes would lead to failure to induce *Atoh1* expression and hair cell fate specification. Deletion of *Eya1* in the differentiating hair cells using *Atoh1-CreER* at E13.5–14.0 fails to activate *Atoh1* and results in the absence of both inner and outer hair cell differentiation in the apex [11], demonstrating the necessity of Eya1 activity for *Atoh1* activation in vivo, which is induced from ~E13.0 and becomes detectable at ~E13.5 [4, 31]. So how do we explain the presence of *Atoh1* expression in the *Six1* CKO mutant cochlea? There are two probable explanations. First, as it takes at least ~6 hours for tamoxifen to induce CreER in the nucleus, it is possible that weak *Atoh1* expression might have already been induced in some precursors before complete removal of *Six1* by tamoxifen administration from E11.5–12.5. Such weak *Atoh1* expression might have been sufficient to induce hair cell differentiation, which would also explain why outer hair cells failed to form and why the number of inner hair cells in the *Six1* CKO decreased to ~53% of that of the wild-type control. Second, there may be functional redundancy with other members of the Six gene family. While *Six4* mice are normal [32], Six4 is coexpressed with Six1 in otic progenitors in the otocyst [13] and in the cranial placodes, and is known to play redundant roles with Six1 in cranial placode development as well as in several other organ systems [33–39]. Thus, it is possible that Six4 activity in the otic epithelial precursors may participate in complex formation with Eya1 and Sox2 to activate *Atoh1* in the prosensory precursors. In support of this idea, we found that coexpression of Eya1 and Six4 in cochlear explants can also induce hair cell fate as efficiently as the combination of Eya1 and Six1 [11]. Besides Six4, weak Six2 expression is detected in the cochlear epithelium [40], where Six5 activity may also exist as mutations in human SIX5 [41], SIX1 [15] or EYA1 [42, 43] cause Branchio-Oto-Renal or Branchio-Oto syndrome. Thus, Six4, Six2 or Six5 in the prosensory progenitor cells may participate in complex formation with Eya1 and Sox2 for initial Atoh1 activation, but this is clearly insufficient to replace Six1 in upregulating Atoh1 in the differentiating hair cells either due to the absence of their expression or low activity in the differentiating hair cells. While the relative ratio of Sox2, Eya1, Six1 and Atoh1 in the precursor cells may be important in specifying hair cells, our data clearly demonstrate that Six1 activity is necessary for specifying hair cell fate in vivo.

The pattern of the outer hair cell defect induced by tamoxifen at later stages between ~E13.0–14.5 provides additional support for Six1 as a key factor in specifying the gradient pattern of hair cell differentiation longitudinally and laterally in the cochlea. In the normal organ of Corti, hair cell differentiation begins in the mid-base and extends not only laterally, but also towards both the basal end and the apex of the cochlea [4]. The longitudinal pattern of hair cell differentiation is also disrupted in the *Six1* CKO, which had no hair cell formation in the basal end (Fig 4B). Thus, Six1 is likely to play a key role in establishing the hair cell developmental program within the auditory sensory epithelial sheet. While detailed characterization of the Six1-regulatory network controlling hair cell development is an ongoing project in the laboratory, our observation of downregulation of *Fgf8* in the *Six1* CKO suggests that Six1 may regulate Fgf8 signaling pathway during differentiation. A key piece of supporting evidence for this has been recently obtained from our ChIP-seq analysis for whole-genome mapping of Six1 in the cochlea, which identified a Six1 peak near the *Fgf8* gene. Characterization of this Six1-bound peak sequence in transgenic mice by cloning upstream of the *Hsp68* minimal promoter driven-LacZ reporter cassette flanked by the H19 insulators has indicated that this enhancer fragment is a cochlear hair-cell specific enhancer (J. Li, E. Loh, J. Xu, T. Zhang, L. Shen and P-X. Xu, manuscript in preparation). While detailed transgenic and mutational analyses of the Six1-binding site within this enhancer of *Fgf8* in vivo are still currently underway, our data suggest that *Fgf8* is a direct in vivo target of Six1 in the cochlea.

In the organ of Corti, Fgf8 is expressed in the inner hair cells and previous studies have suggested that Fgf8 may act through its receptor Fgfr3 to regulate pillar cell development [25, 44, 45]. In *Six1* CKO, inner pillar cells marked by p75 are present (Fig 8A and 8B), but pillar cells that are negative for S100A appear to be missing (Fig 5A). Since the inner pillar cell lies next to the inner hair cell, we speculate that outer pillar cell is more sensitive to the Fgf8 levels, and a decrease in the effective range of Fgf8 signaling due to reduced Fgf8 levels in the inner hair cells in *Six1* CKO mutant might result in the lack of outer pillar cell formation. In addition to *Fgf8*, Six1-bound regions associated with *Fgfr1* have also been identified by Six1 ChIP-seq analysis (J. Li, E. Loh, J. Xu, T. Zhang, L. Shen and P-X. Xu, manuscript in preparation). Given the similarity of the phenotype between the *Six1* CKO and the *Fgfr1* mutants, which range from missing the outermost row of outer hair cells in mildly hypomorphic *Fgfr1* mutant to only residual numbers of inner hair cells in the most severe mutants, *Six1* may also regulate Fgfr1 signaling in the organ of Corti.

One intriguing finding of this study is the high level of Sox2 expression in the *Six1* CKO hair cells. While Sox2 is expressed in type II hair cells in the adult mouse utricle [46], it is absent from auditory hair cells. Sox2 is known to play a direct role in establishing the prosensory domain within the cochlea, but Sox2 alone is unable to induce *Atoh1* expression [8], as it interacts with Eya1 and Six1 to regulate the initiation of *Atoh1* expression [11]. However, following the onset of Atoh1 expression in the hair cell precursors, Sox2 levels become downregulated in differentiating hair cells, and an antagonistic interaction between Sox2 and Atoh1 was suggested to play a role in this downregulation [8]. In *Six1* CKO cochlea, although the expression level of Atoh1 appears to be lower than normal, it is sufficient to promote subsequent hair cell differentiation to generate Myo7a^+^ hair cells even in the presence of high levels of Sox2. Thus, the capacity of endogenous Atoh1 to direct the hair cell differentiation program does not depend on its ability to downregulate Sox2. In agreement with this view, forced expression of Atoh1 in cochlear epithelium in young mice is able to induce Sox2^+^ cells to become ectopic Myo7a^+^ hair cells in the GER [47], but Sox2 levels appear to drop in Myo7a^+^ ectopic hair cells as their differentiation advances. Although detailed Six1 expression in postnatal cochlea has not been studied, the GER cells in young animals soon after birth are likely to retain some levels of Six1, which is widely expressed in the cochlear epithelium at birth (Fig 3). Our data show that in the absence of Six1, Sox2 does not appear to inhibit Atoh1 function in the differentiating hair cells. In the presence of Six1, deletion of Atoh1 in hair cells at later stages does not lead to upregulation of Sox2. Therefore, the antagonistic effect between Sox2 and Atoh1 is likely to be indirect and mediated through other factors, which may vary among different types of hair cells in the inner ear. This would explain why there are Sox2^+^ type II hair cells in the utricle and why a subset of Atoh1-induced high Sox2^+^ ectopic hair cells exist in the cochlea [47]. While future studies are necessary to determine how many factors are involved in Sox2 downregulation in the auditory hair cells and how Six1 works together with them to repress Sox2 expression, our finding of high levels of Sox2 in Atoh1^+^ hair cells in *Six1* CKO cochlea uncovers a previously unknown function of Six1 in regulating the spatiotemporal pattern of Sox2 during the differentiation of the organ of Corti.

Our data show that Six1 is essential not only for hair cell fate induction, but also for proper patterning of the postmitotic precursor cells in the sensory epithelium. The precursor cells undergo rearrangements through both mediolateral and radial intercalation to achieve extension and establish the mosaic structure between hair cells and supporting cells [4, 48]. These processes require adhesion changes that allow cells to move and maintain adhesion, and cadherins are known to control differential adhesive properties of cells during morphogenesis [49]. In the cochlea, the adhesion junction proteins E-cadherin and N-cadherin at the cell membrane mark a sharp boundary between the inner and outer hair cells and a direct involvement of these proteins in convergent extension in the cochlea has been shown recently [28]. In *Six1* CKO cochlea, the sharp border formed by the expression of N-cadherin or E-cadherin is disrupted and their expression is expanded to all cells in the organ of Corti. Such alterations in cadherin distribution are likely to lead to cellular morphological alterations. These data together support a role for Six1 in establishing the mosaic structure between hair cells and supporting cells in the organ of Corti.

Lastly, it is worth mentioning that *Six1* gene dosage may have differential effect on the development between inner and outer hair cells, as the hypomorphic *Cwe/+* heterozygous mice have extra inner hair cells in the apex of the cochlea, while their outer hair cells appear unaffected [14]. Similar phenotype also occurs in *Eya1*^*+/-*^ [50] and *Eya1*^*CreER*^ mice (Fig 6A). Thus, it is possible that Six1 may be required at a certain level at specific time points in cochlear development to regulate a different set of genes between inner and outer hair cell precursors that are particularly sensitive to the level of Six1. Six1 may mediate organ of Corti formation through Fgf signaling, including Fgf8 signaling. While allelic series of *Six1* will provide insight into its dosage effect on Fgf signaling and inner ear development, whole-genome mapping of Six1-DNA interactions and identification of its direct targets at different developmental stages will be necessary toward understanding how sensory progenitor cells use Six1 to create precise patterns of gene expression and cell differentiation to shape and generate a functional organ of Corti for hearing in the mammalian inner ear.

## Methods

### Ethics statement

All animal protocols were approved by Animal Care and Use Committee of the Icahn School of Medicine at Mount Sinai (protocol #06–0807).

### Animals, genotyping and tamoxifen administration

*Six1*^*Flox*^ [17], *Eya1*^*CreERT2*^ [11], *Sox2*^*CreERT2*^ [19], and *Atoh1*^*Flox*^ (JAX # 008681) mice were maintained on a 129/Sv and C57BL/6J mixed background at the Icahn School of Medicine at Mount Sinai Animal Facility.

Mice were bred using timed mating, and noon on the day of vaginal plug detection was considered as E0.5. For induction of the CreERT2 protein, tamoxifen (Sigma, T5648) was dissolved in corn oil (Sigma, C8267) and administrated (1.5 mg/10 g body weight) by oral gavage. Observed variations among *Six1* mutants is likely due to pre-existing developmental variation between embryos when tamoxifen was given.

### Histology, immunohistochemistry and in situ hybridization (ISH)

Histological examination, whole-mount and section immunostaining and ISH were carried out according to standard procedures. Briefly, inner ears were fixed in 4% paraformaldehyde (PFA) for 1 hr at 4°C, dehydrated, and embedded in wax. Paraffin sections were generated at 6 μm. For ISH, tissues were fixed overnight. We used five embryos for each genotype at each stage for each probe and the result was consistent in each embryo.

Primary antibodies: anti-Sox2 (PA1-094, Thermo Fisher), -Myo7A (25–6790, Proteus and 138-1-s, DSHB), -Six1 (HPA001893, Sigma), -Atoh1 (Math1-s, DSHB), -p27^kip1^ (554069, BD Pharmingen), -Calretinin (MA5-14540, Thermo Fisher), -p75^NTR^ (#07–476, EMD Millipore), -N-cadherin (610921, BD Bioscience), -E-cadherin (U3254, Sigma), -S100A (ab11428, Abcam), -GLAST (ab416, Abcam), -Pou4f3 (sc-81980, Santa Cruz), -Prox1 (AB5475, Millipore), -Acetylated tubulin (T7451, Sigma), -Cy3-, Cy2-, Cy5- and FITC-conjugated secondary antibodies were used. Alexa Fluor 488 or 350-conjugated phalloidin (A12379 and A22281, Life technologies) were used for actin staining. Hoechst 3342 was used for nuclear staining.

### EdU and TUNEL assays

The EdU assay was performed using a kit (catalog no. C10640, Life Technologies) following the manufacturer’s instructions. EdU was co-injected with tamoxifen at 9 am of E11.5 and embryos were harvested at noon of E14.5. EdU was also injected at noon of E14.5 embryos following tamoxifen treatment at 9 am of E11.5 and embryos were harvested at noon of E17.5. The TUNEL assay was performed using the Apop Tag kit for in situ apoptosis fluorescein detection (catalog no. NC9815837, Millipore) following the manufacturer’s instructions.

### Reverse transcription and real-time PCR

Whole inner ears collected from E15.5 or E17.5 embryos were divided into two parts with forceps and the cochlear parts, which also contained the spiral ganglion, were used for total RNA extraction using Trizol Reagents (Invitrogen). Total RNAs were treated with RNase-Free DNase Set (QIAGEN) and then used for reverse transcription using a SuperScript IV Reverse Transcriptase (Thermo Fisher Scientific) for first-Strand cDNA Synthesis. Gene specific primers and SYBR Green Master Mix (Applied Biosystems) were used for PCR amplification using the Applied Biosystems StepOnePlus Real-Time PCR Systems. Expression levels of each transcript were normalized using β-actin as an internal control. Each set of experiments was repeated three times, and the DDCT relative quantification method was used to evaluate quantitative variation. Two-tailed Student's t test was used for statistical analysis. Primers used are as follows: for *Atoh1*, forward primer-5’-GCTTATCCCCTTCGTTGAACT-3’ and reverse primer-5’-TGCTATCCAGGAGGGACAGTTCTG-3’; for *Fgf8*, forward primer-5’-ACGACATTCCACGAGCCGCGTC-3’ and reverse primer-5’-GAAGGGTCGGTCCTCGTGTCCCT-3’; and for *β-actin*, forward primer 5’-AACGGCTCCGGCATGTGCAAAG-3’ and reverse primer 5’-ACACGCAGCTCATTGTAGAAG-3’.

### Cell counts and spatial calibration

EdU-incorporated Sox2^+^ prosensory progenitor cells in the floor of the cochlear epithelium were counted in basal, medial and apical turn of the entire cochlea. Values represent average number of EdU^+^Sox2^+^ cells (±standard deviations) per section (6 μm) or per cochlea. Width and height of the Sox2^+^ or p27^kip1+^ domain at E14.5 were measured on sections (height at 6 μm/section) for spatial calibration using Image J software (NIH). 15 sections per cochlea and 2 cochleae for each sample were measured. Two-tailed Student's t test was used for statistical analysis.

## Supporting information

S1 Fig Six1 expression is lost in the hair cell precursors in *Six1* CKO cochlea (given tamoxifen at E11.5–12.5) using either *Eya1^CreERT2^* or *Sox2^CreERT2^* as a deletor. (A) Lineage tracing using *R26R*^*LacZ*^ reporter confirmed that one dose of tamoxifen administration at E11.5 induced *Eya1*^*CreERT2*^-lineage traced cells in the GER and all cells in the organ of Corti, including some Henson’s cells at P0. (B) Six1 (red)/p27Kip1 (green)costaining showing Six1 reduction in the sensory region in *Eya1*^*CreER*^*;Six1*^*fl/fl*^. (C-E) Six1 (red) and Myo7a (green) section staining from E18.5 wild-type (C), *Eya1*^*CreERT2*^*; Six1*^*fl/fl*^ (D) and *Sox2*^*CreERT2*^*; Six1*^*fl/fl*^ cochlea (E). Scale bars: 30 μm.
(PDF)

S2 Fig Smaller inner ears and a slight delay in the development of the prosensory progenitors in *Six1* CKO given tamoxifen at E11.5–12.5. (A,B) *Six1* CKO mutants have smaller inner ears, compared to *Eya1*^*CreER*^ or wild-type littermates at E14.5 (A) and E17.5 (B). (C) Cochlear sections stained with p27^Kip1^ (green) and EdU (red) from E17.5 *Eya1*^*CreER*^ or *Six1* CKO littermate embryos injected with EdU at E14.5. Scale bar: 30 μm.
(PDF)

S3 Fig Abnormal apoptosis was not significantly increased in *Six1* CKO cochlea between E12.5-E14.5 given tamoxifen at E11.5-E12.5. TUNEL assay on sections from E14.5 wild-type (A,B) or *Six1* CKO (*Eya1*^*CreERT2*^*;Six1*^*fl/fl*^) littermate cochlea (C,D) or E13.5 *Eya1*^*CreER*^ (E) or *Six1* CKO littermate cochlea (F) showing apoptotic cells in the cochlear epithelium (red). (G) Statistical analysis showing average number of apoptotic cells in the floor of cochlear epithelium per section (6 μm) at E12.5 (*p* = 0.023), E13.5 (*p* = 0.09) and E14.5 (*p* = 0.07). Scale bars: 100 μm.
(PDF)

S4 Fig Largely reduced utricular and saccular macula with fewer hair cells and no hair cells in crista ampullaris in all three semicircular canals. (A-F) Myo7a (green) and Sox2 (red) staining on sections of utricle (A,B), saccule (C,D) and crista (E,F) from E18.5 wild-type or *Eya1*^*CreERT2*^*; Six1*^*fl/fl*^ cochlea given tamoxifen at E11.5 and E12.5. Scale bars: 100 μm.
(PDF)
