# Supplementary figures and images for: Six1 is essential for differentiation and patterning of the mammalian auditory sensory epithelium
Source: PLoS Genet. 2017 Sep 11;13(9):e1006967. doi: 10.1371/journal.pgen.1006967 (PMC5593176; doi:10.1371/journal.pgen.1006967)

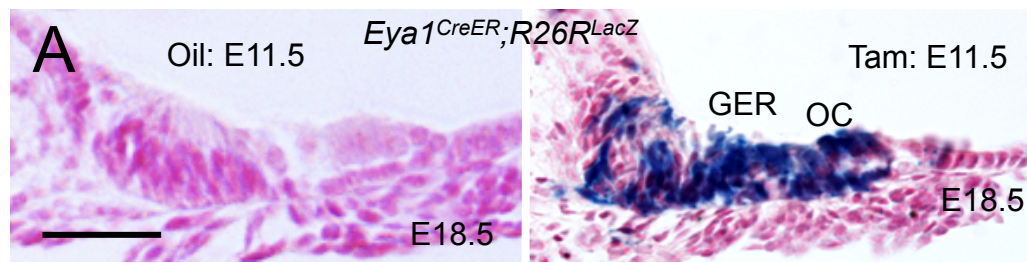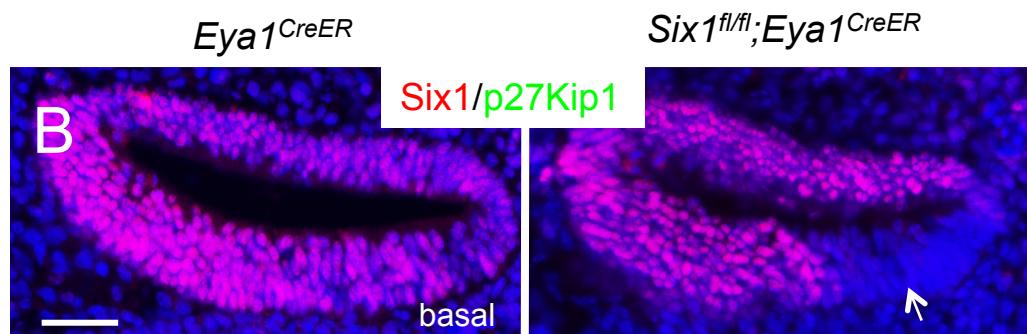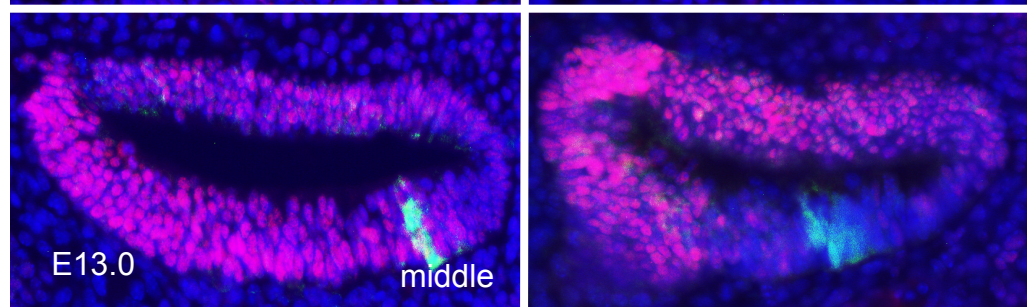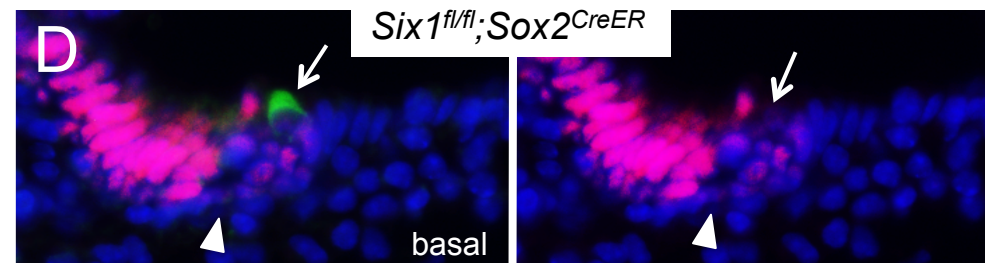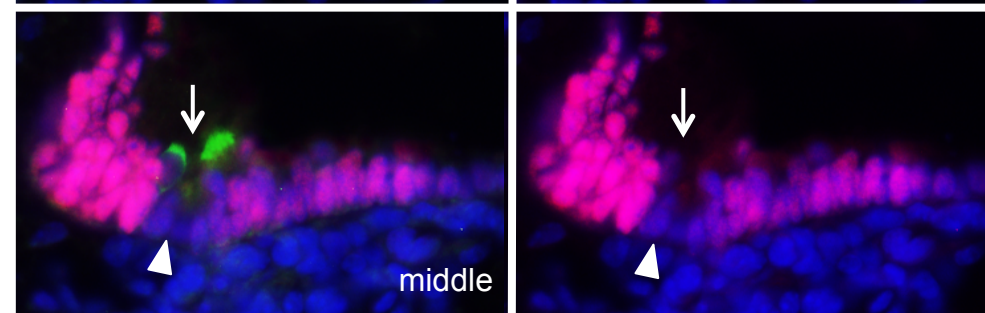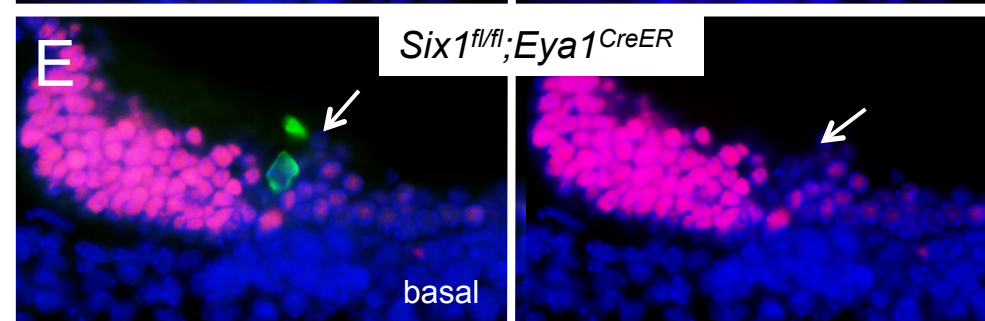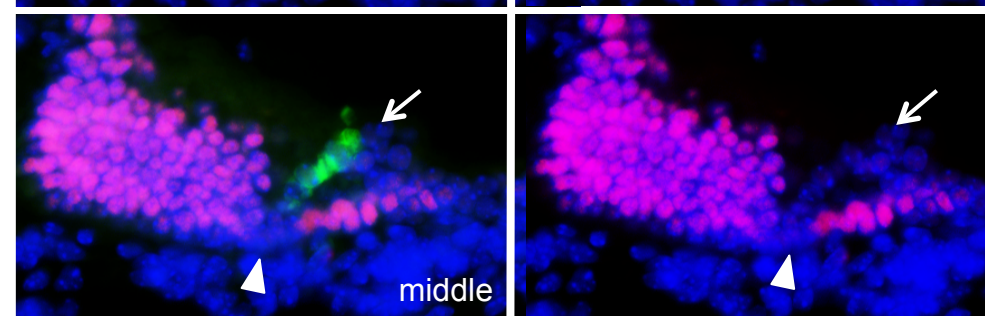

Supplement: S1 Fig — (A) Lineage tracing using R26RLacZ reporter confirmed that one dose of tamoxifen administration at E11.5 induced Eya1CreERT2-lineage traced cells in the GER and all cells in the organ of Corti, including some Henson’s cells at P0. (B) Six1 (red)/p27Kip1 (green)costaining showing Six1 reduction in the sensory region in Eya1CreER;Six1fl/fl. (C-E) Six1 (red) and Myo7a (green) section staining from E18.5 wild-type (C), Eya1CreERT2; Six1fl/fl (D) and Sox2CreERT2; Six1fl/fl cochlea (E). Scale bars: 30 μm. (PDF) [file pgen.1006967.s001.pdf]

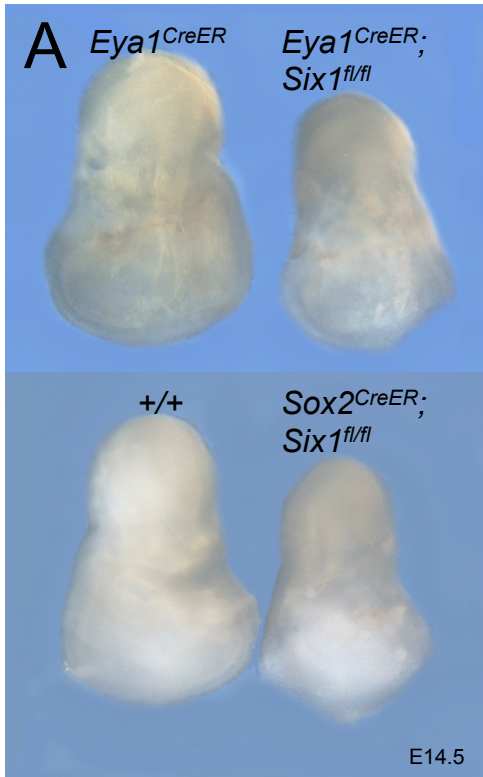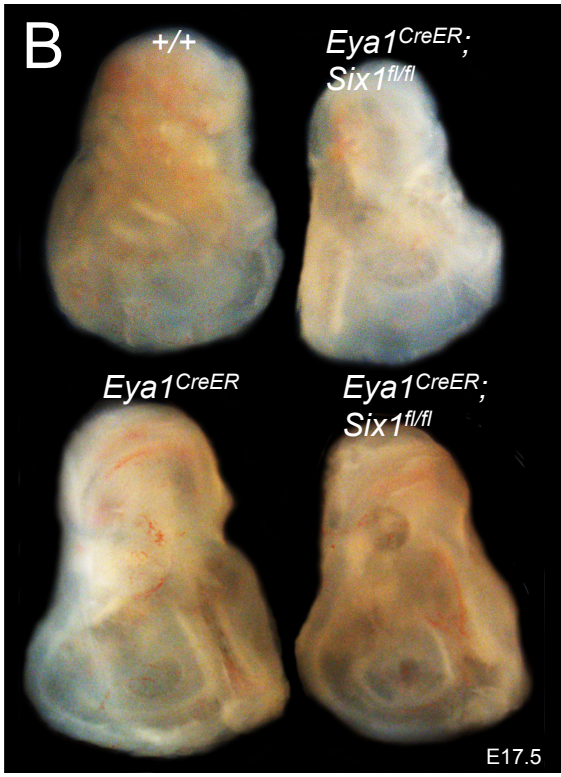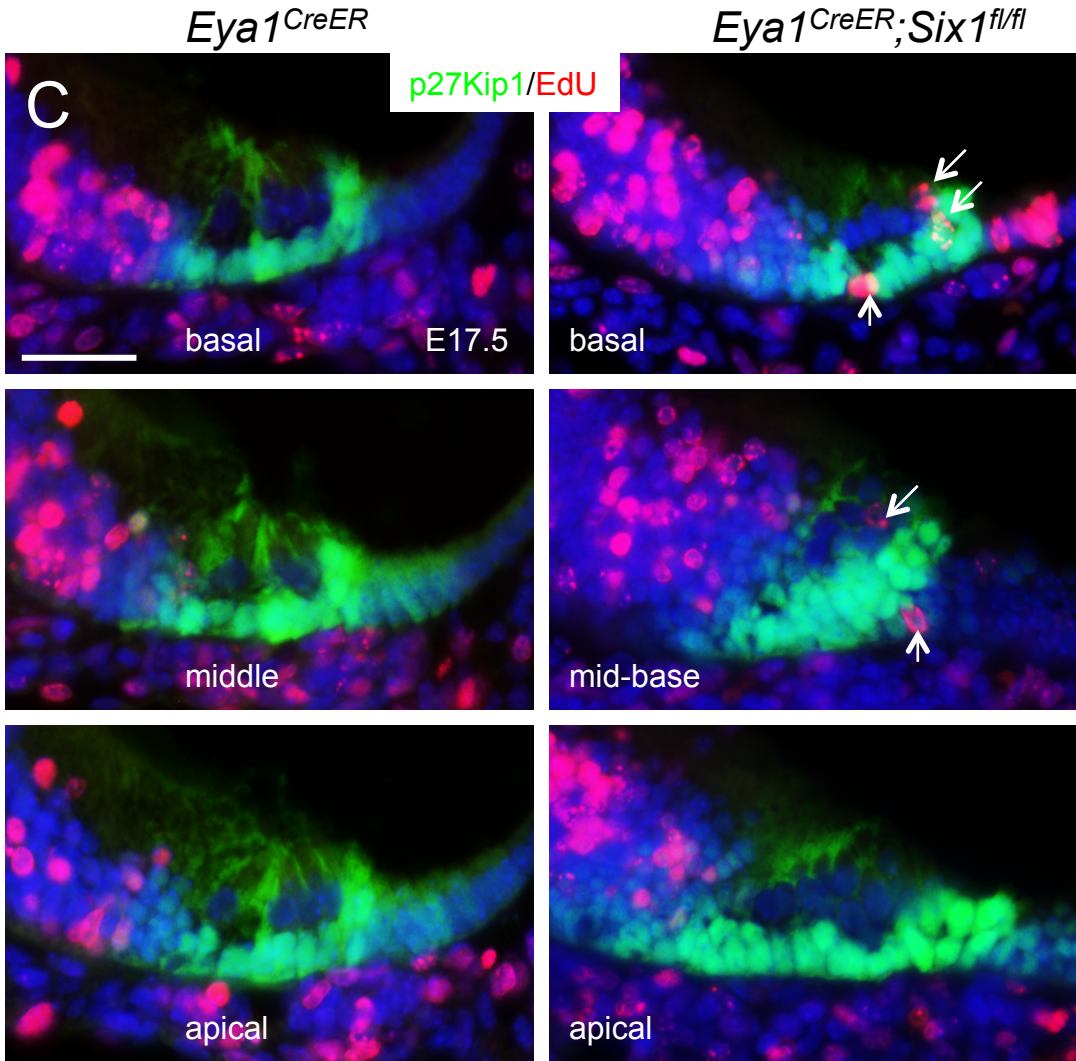

Supplement: S2 Fig — (A,B) Six1 CKO mutants have smaller inner ears, compared to Eya1CreER or wild-type littermates at E14.5 (A) and E17.5 (B). (C) Cochlear sections stained with p27Kip1 (green) and EdU (red) from E17.5 Eya1CreER or Six1 CKO littermate embryos injected with EdU at E14.5. Scale bar: 30 μm. (PDF) [file pgen.1006967.s002.pdf]

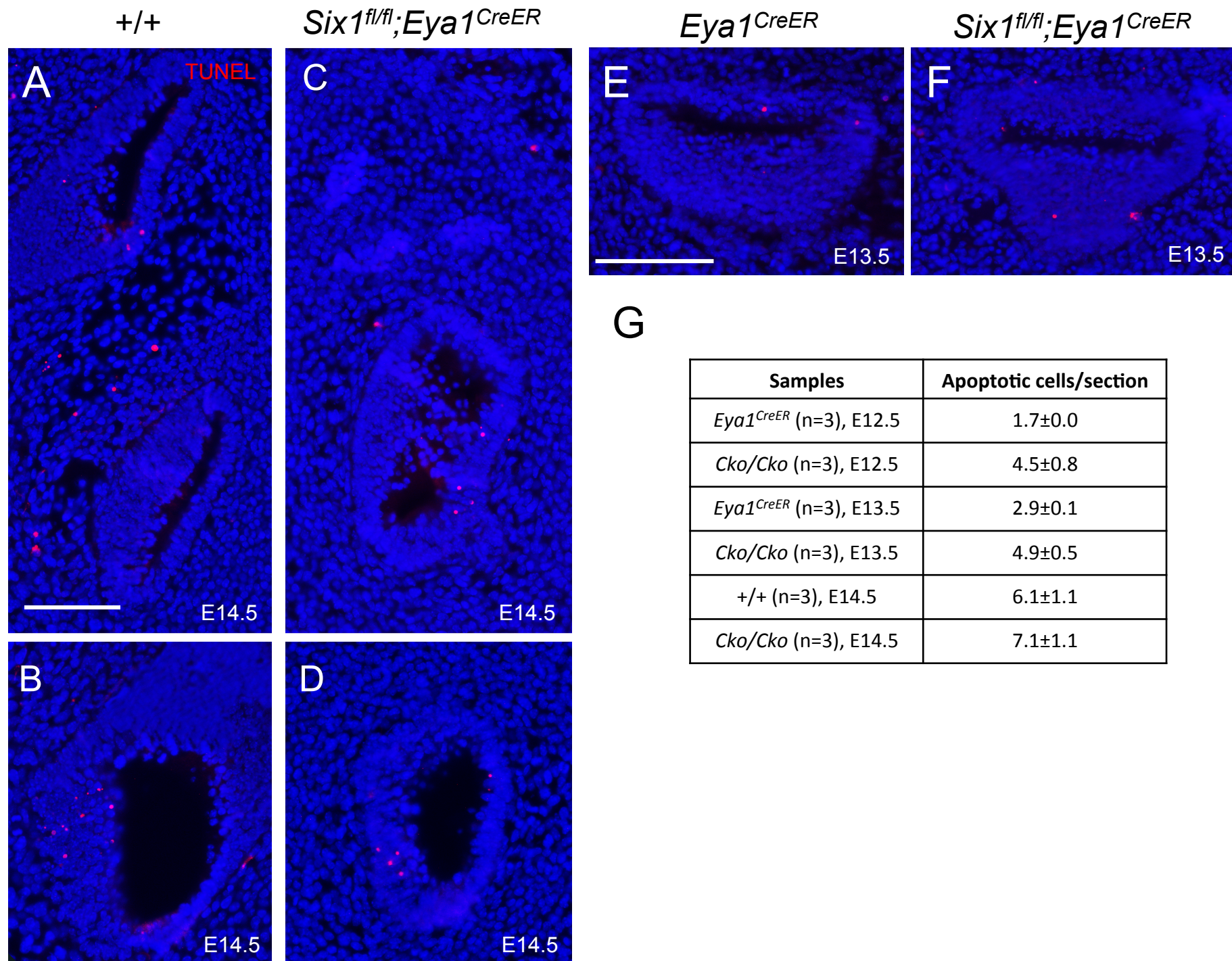

Supplement: S3 Fig — TUNEL assay on sections from E14.5 wild-type (A,B) or Six1 CKO (Eya1CreERT2;Six1fl/fl) littermate cochlea (C,D) or E13.5 Eya1CreER (E) or Six1 CKO littermate cochlea (F) showing apoptotic cells in the cochlear epithelium (red). (G) Statistical analysis showing average number of apoptotic cells in the floor of cochlear epithelium per section (6 μm) at E12.5 (p = 0.023), E13.5 (p = 0.09) and E14.5 (p = 0.07). Scale bars: 100 μm. (PDF) [file pgen.1006967.s003.pdf]

Supplementary Fig. 4

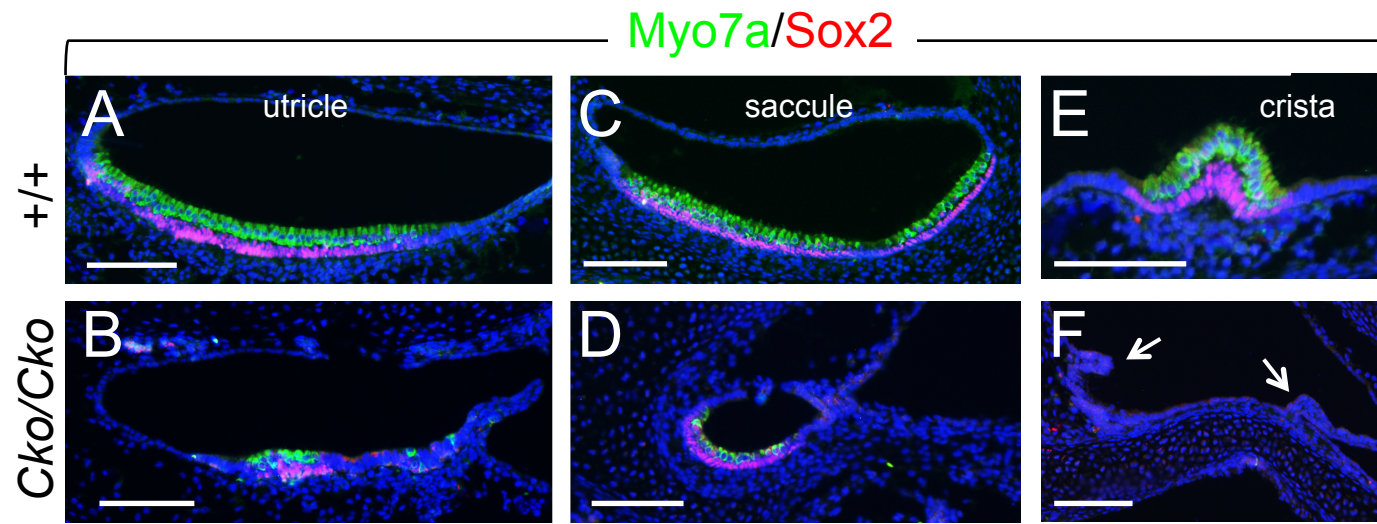

Supplement: S4 Fig — (A-F) Myo7a (green) and Sox2 (red) staining on sections of utricle (A,B), saccule (C,D) and crista (E,F) from E18.5 wild-type or Eya1CreERT2; Six1fl/fl cochlea given tamoxifen at E11.5 and E12.5. Scale bars: 100 μm. (PDF) [file pgen.1006967.s004.pdf]
